# Supplementary material for: Microglia promote glioblastoma via mTOR‐mediated immunosuppression of the tumour microenvironment
Source: EMBO J. 2020 Jun 22;39(15):e103790. doi: 10.15252/embj.2019103790 (PMC7396846; doi:10.15252/embj.2019103790)
Supplement: Supplementary file 1 — Appendix [file EMBJ-39-e103790-s001.pdf]

Table of Content

Appendix Figure S1
Appendix Figure S2
Appendix Figure S3

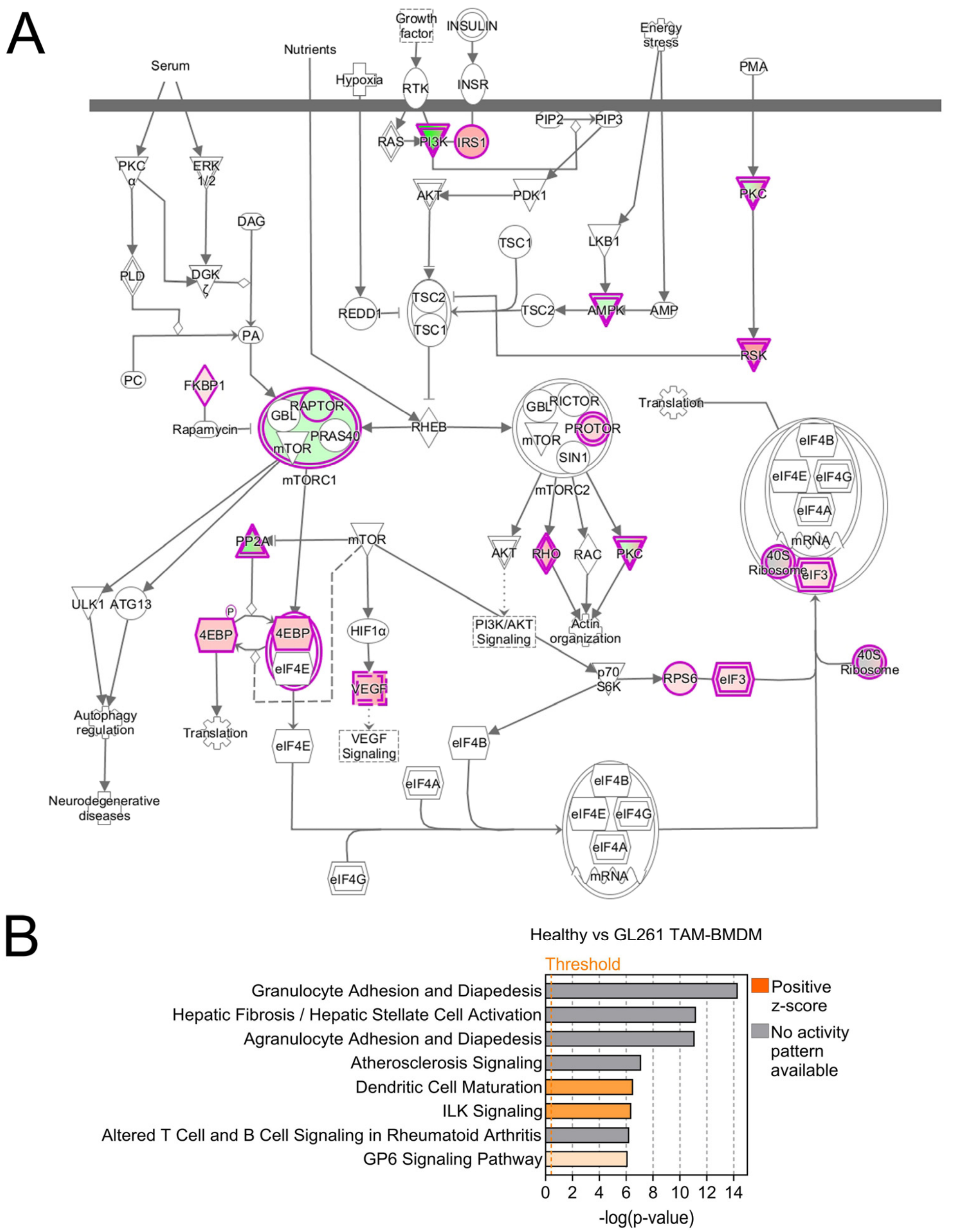

Appendix Figure S1

(A) Schematic representation of the mTOR pathway with genes deregulated in GL261 TAM-MG versus healthy microglia. Pink circles indicate deregulated expression of the gene, which when coloured green is downregulated and in pink upregulated. (B) Top-most deregulated canonical pathways in GL261 TAM-BMDM versus healthy blood monocytes, as identified by the IPA software. Threshold indicates $p \leq 0.05$. Z-score indicating the orientation of the deregulation.

A

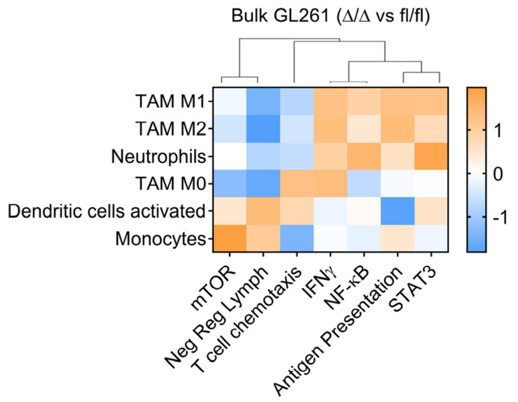

Appendix Figure S2

(A) Heatmap of differential correlation analysis between CIBERSORT cell fractions and signatures enrichment scores (ssGSEA) for myeloid cells in *Cx3cr1-Rheb1*$^{Δ/Δ}$ (n=3) versus *Rheb1*$^{fl/fl}$ (n=3) GL261 tumours.

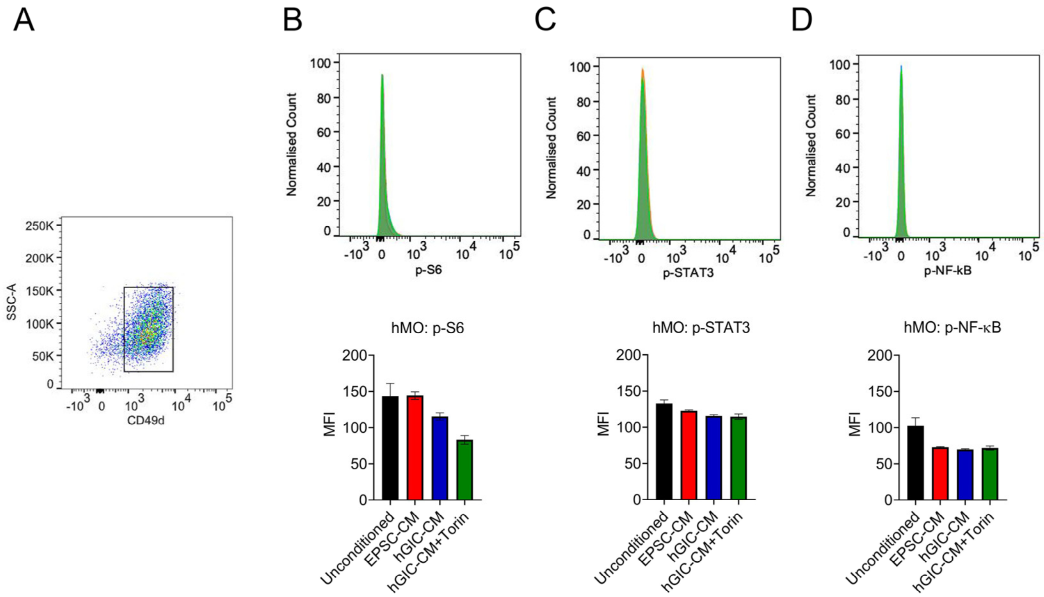

Appendix Figure S3

(A) Representative flow cytometry plot of CD49d expression in the healthy blood-derived monocytes/macrophages (on the right). (B) Representative histogram and quantification of the MFI levels of p-S6, (C) p-STAT3 and (D) p-NF-κB (p-P65) in conditioned human monocytes/macrophages (hMO; n=3, mean ±SEM, One-Way ANOVA Tukey tests – on the right).
