# Supplementary material for: Microglia promote glioblastoma via mTOR‐mediated immunosuppression of the tumour microenvironment
Source: EMBO J. 2020 Jun 22;39(15):e103790. doi: 10.15252/embj.2019103790 (PMC7396846; doi:10.15252/embj.2019103790)
Supplement: Supplementary file 2 — Expanded View Figures PDF [file EMBJ-39-e103790-s002.pdf]

Expanded View Figures

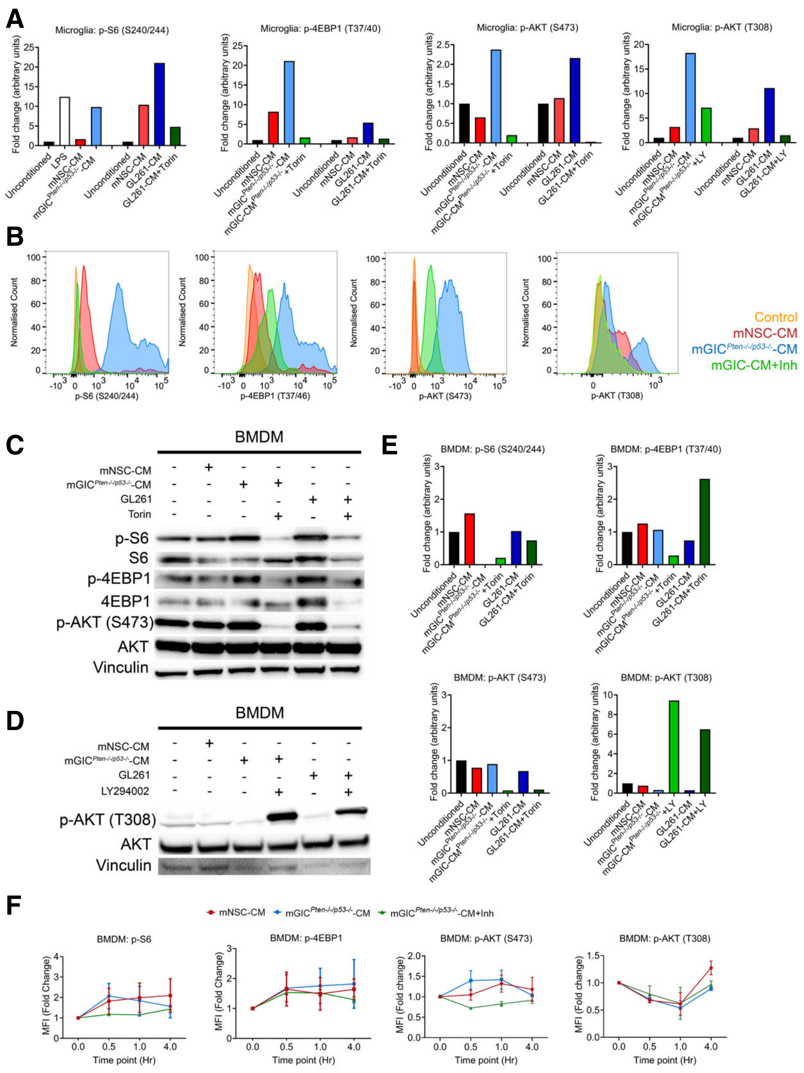

Figure EV1. Microglia and BMDM are differently conditioned by mGIC-secreted factors *in vitro*.

A Quantification of immunoblotting analysis from conditioned microglia whole cell lysates at 4 h incubation for p-S6, p-4EBP1 and p-AKT S473 and at 0.5 h incubation for p-AKT T308. Data normalised to non-phosphorylated protein and to vinculin.

B Representative flow cytometry plots of microglia under different culture condition at 4 h incubation for p-S6, p-4EBP1 and p-AKT S473 and at 0.5 h incubation for p-AKT T308.

C–E Signalling was analysed in conditioned BMDM by immunoblotting of whole cell lysate collected at 4 h for p-S6, p-4EBP1 and p-AKT S473 (C) and at 0.5 h incubation for p-AKT T308 (D), which was quantified by normalisation with non-phosphorylated protein and vinculin (E).

F Flow cytometry analysis was carried out in BMDM for p-S6, p-4EBP1, p-AKT S473 and p-AKT T308. Each treatment was normalised to unconditioned control (n = 3; mean ± SEM; two-way ANOVA Tukey test).

Source data are available online for this figure.

 © 2020 The Authors

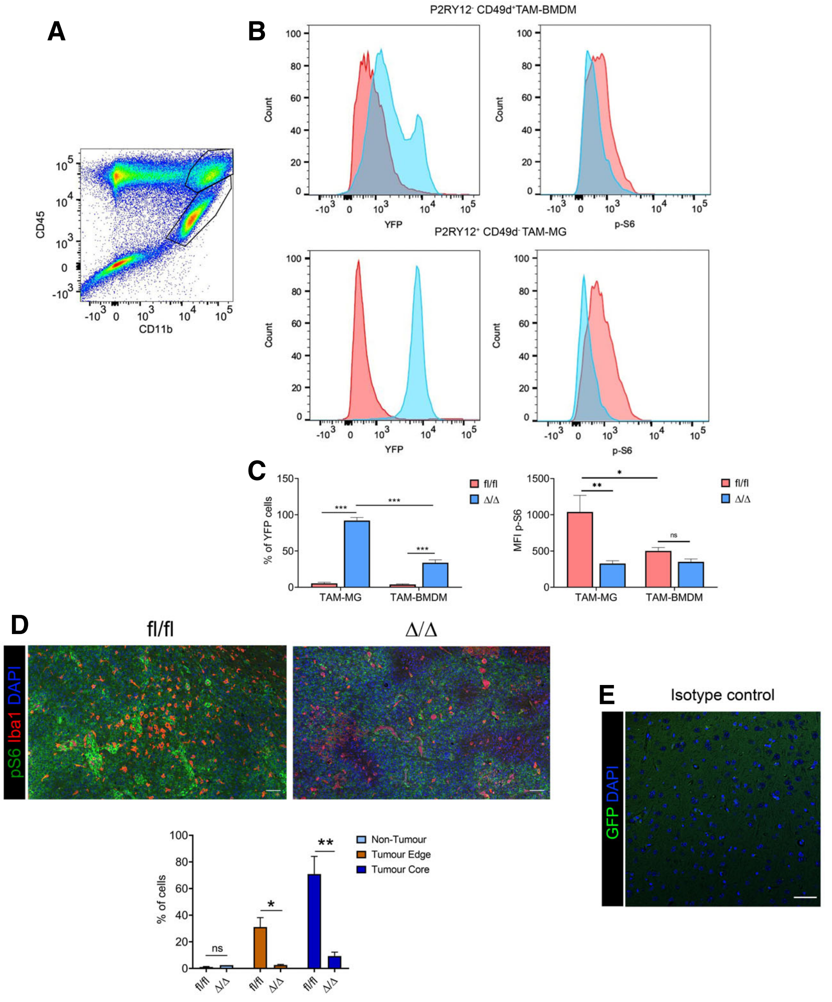

Figure EV2. ***Cx3cr1-Rheb1*$^{\Delta/\Delta}$ mice display inhibition of mTORC1 signalling in GL261 TAM.**

A Representative flow cytometry plot for the expression of CD45 and CD11b in GL261 tumour, with CD45^{high} CD11b^{+} TAM-BMDM (top gate) and CD45^{low} CD11b^{+} TAM-MG (lower gate).

B Representative flow cytometry plot of YFP (left) and p-S6 (right) levels in TAM-BMDM (top) and TAM-MG (bottom) in *Cx3cr1-Rheb1*$^{\Delta/\Delta}$ (blue) versus *Rheb1*$^{fl/fl}$ (red) GL261 tumours.

C Percentage of TAM-MG and TAM-BMDM expressing YFP in *Cx3cr1-Rheb1*$^{\Delta/\Delta}$ (n = 6) and *Rheb1*$^{fl/fl}$ (n = 6) mice. MFI levels of p-S6 in P2RY12^{+} CD49d^{-} TAM-MG and P2RY12^{-} CD49d^{+} TAM-BMDM in *Rheb1*$^{fl/fl}$ (n = 6) compared to *Cx3cr1-Rheb1*$^{\Delta/\Delta}$ (n = 6) GL261 tumours (mean ± SEM; two-way ANOVA Tukey test).

D Staining for Iba1, p-S6 and DAPI in *Rheb1*$^{fl/fl}$ (n = 3) and *Cx3cr1-Rheb1*$^{\Delta/\Delta}$ (n = 2) tumour tissue (top) and percentage of Iba1^{+} cells co-expressing p-S6 in the three defined regions (bottom) (mean ± SEM; two-way ANOVA Tukey test). Scale bar is 100 µm.

E Isotype control stain demonstrating quenching of fluorescence from reporter gene (scale bar 50 µm).

Data information: $*P \leq 0.05$, $**P \leq 0.01$, $***P \leq 0.001$, ns non-significant.

© 2020 The Authors

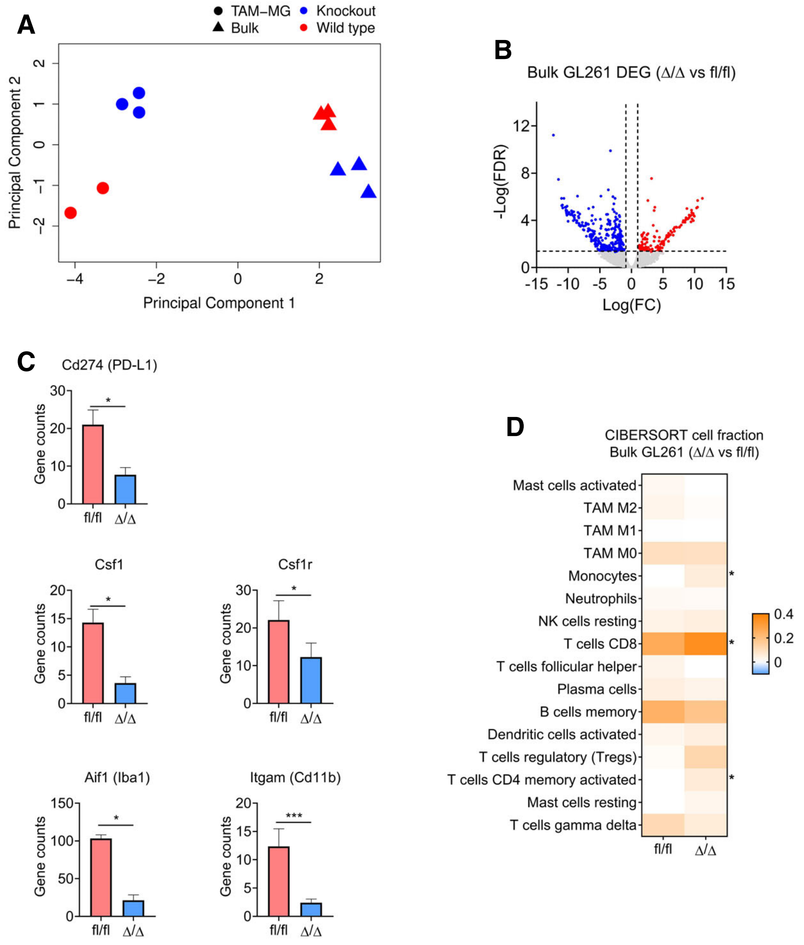

Figure EV3. Genetic inhibition of mTORC1 signalling in TAM affects the immune landscape of GL261 tumours.

A Principal component analysis of bulk (triangle) and TAM-MG (circle) RNA-Seq samples from *Rheb1*$^{fl/fl}$ (red) and *Cx3cr1-Rheb1*$^{\Delta/\Delta}$ (blue) Gl261 tumours.

B Volcano plot of differentially expressed genes between *Rheb1*$^{fl/fl}$ (n = 3) and *Cx3cr1-Rheb1*$^{\Delta/\Delta}$ (n = 3) GL261 tumours. Red and blue points mark genes with significantly increased or decreased expression, respectively (FDR $\leq$ 0.05). The x-axis shows expression log fold changes (FC), and the y-axis shows the −log of the false discovery rate (FDR).

C Expression levels (TPM) of Csf1r, Csf1, Aif1 (Iba1), Itgam (Cd11b) and Cd274 (PD-L1) in *Cx3cr1-Rheb1*$^{\Delta/\Delta}$ (n = 3) versus *Rheb1*$^{fl/fl}$ (n = 3) (mean ± SEM, likelihood ratio test in edgeR). *FDR $\leq$ 0.05, ***FDR $\leq$ 0.001.

D CIBERSORT cell fractions calculated from the TPM of *Cx3cr1-Rheb1*$^{\Delta/\Delta}$ (n = 3) versus *Rheb1*$^{fl/fl}$ (n = 3; mean ± SEM; two-way ANOVA Tukey test). *$P \leq 0.05$.

© 2020 The Authors

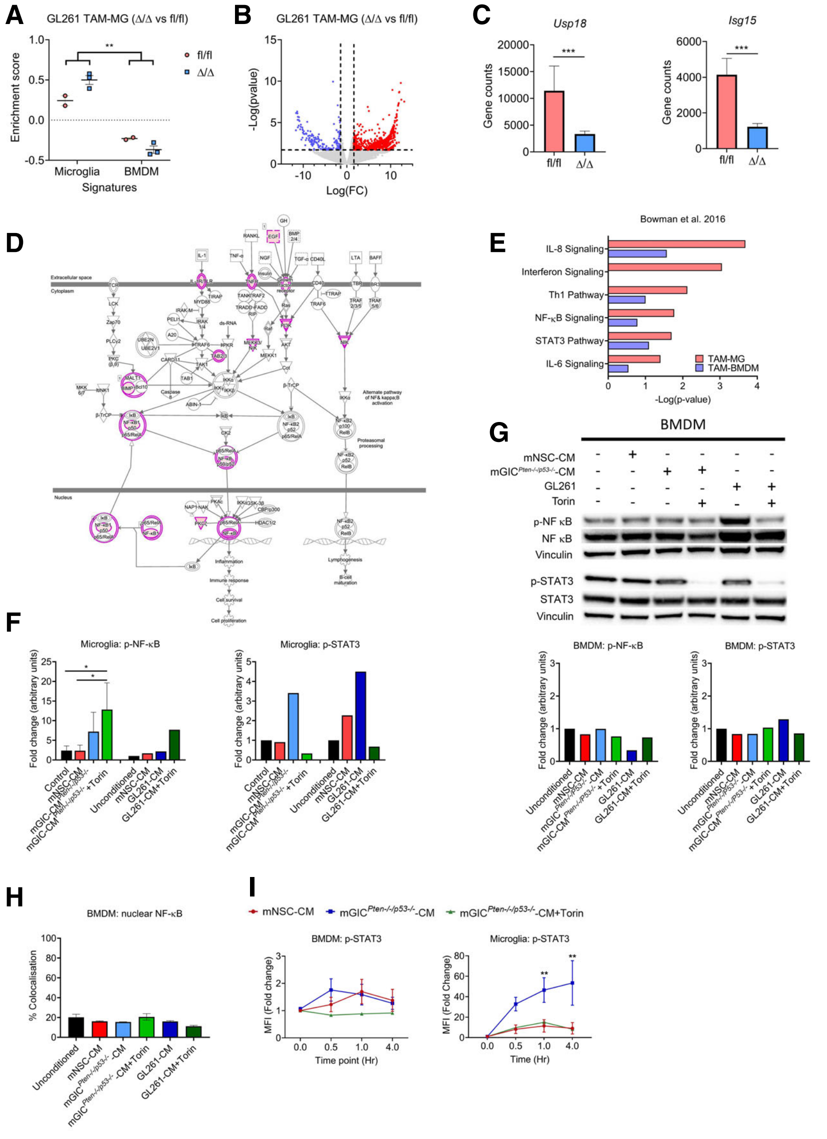

Figure EV4. Tumour-conditioned microglia but not BMDM display mTOR-dependent increased STAT3 and decreased NF-κB signalling.

A ssGSEA enrichment scores from TAM-MG and TAM-BMDM signature in *Rheb1*$^{fl/fl}$ (n = 2) and *Cx3cr1-Rheb1*$^{\Delta/\Delta}$ (n = 3) RNA-Seq from TAM-MG of GL261 tumours (mean ± SEM; two-way ANOVA Tukey tests).

B Volcano plot of differential expression analysis of genes between *Rheb1*$^{fl/fl}$ (n = 2) and *Cx3cr1-Rheb1*$^{\Delta/\Delta}$ (n = 3) TAM-MG. Red and blue points mark genes with significantly increased or decreased expression, respectively ($P \leq 0.02$). The x-axis shows expression log fold changes (FC), and the y-axis shows the −log of the false discovery rate (P-value).

C Expression levels of Isg15 and Usp18 in *Cx3cr1-Rheb1*$^{\Delta/\Delta}$ (n = 3) versus *Rheb1*$^{fl/fl}$ TAM-MG (n = 2) as calculated from the DEG analysis (mean ± SEM, likelihood ratio test in edgeR).

D Schematic representation of the NF-κB pathway with genes deregulated in *Cx3cr1-Rheb1*$^{\Delta/\Delta}$ versus *Rheb1*$^{fl/fl}$ TAM-MG. Pink circles indicate deregulated expression of the protein, which when coloured in pink upregulated.

E Deregulated canonical pathways in GL261 TAM-MG and TAM-BMDM, as identified by the IPA software between *Cx3cr1-Rheb1*$^{\Delta/\Delta}$ and *Rheb1*$^{fl/fl}$ TAM-MG.

F Quantification of immunoblotting analysis from conditioned microglia whole cell lysates at 4 h incubation for p-NF-κB (p-P65) (n = 3) and p-STAT3 (n = 1), normalised to non-phosphorylated protein and vinculin (mean ± SEM; one-way ANOVA Tukey tests).

G Signalling was analysed in conditioned BMDM by immunoblotting of whole cell lysates collected at 4 h for p-NF-κB (p-P65) and p-STAT3, which was normalised to non-phosphorylated protein and vinculin.

H Quantification of nuclear translocation of NF-κB (P65) from immunofluorescence staining. Unit represent the % of voxels in the NF-κB channel colocalised with DAPI (n = 3; mean ± SEM; one-way ANOVA Tukey tests).

I Flow cytometry analysis of p-STAT3 in conditioned microglia and BMDM (n = 3; mean ± SEM; two-way ANOVA Tukey tests).

Data information: $^*P \leq 0.05$, $^{**}P \leq 0.01$, $^{***}P \leq 0.001$.

© 2020 The Authors

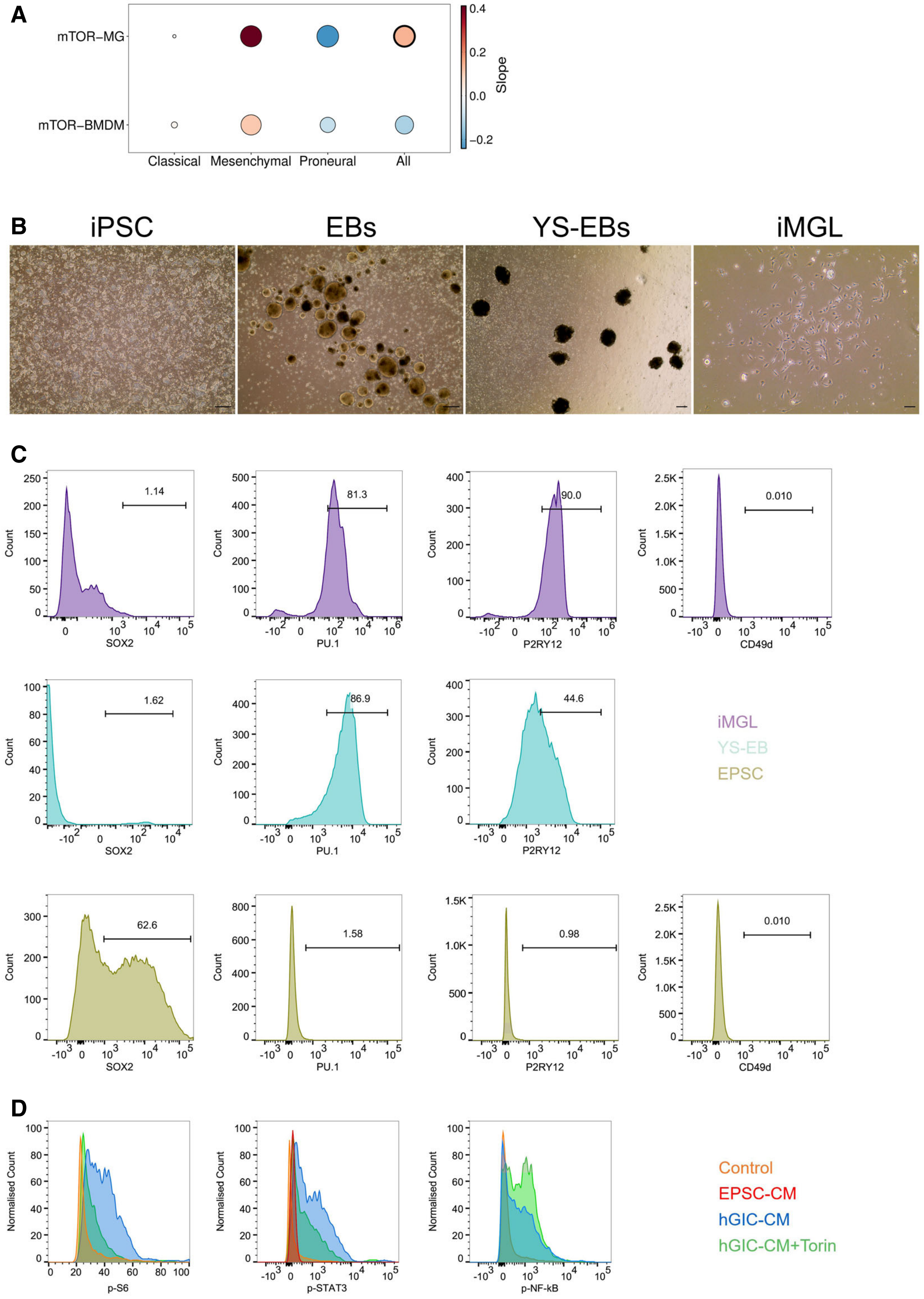

Figure EV5.

 © 2020 The Authors

◀ **Figure EV5. STAT3 and NF-κB signalling are regulated by mTOR activity in TAM-MG in human glioblastoma.**

A Correlation between ssGSEA enrichment scores for the mTOR signature versus TAM-MG or TAM-BMDM signatures in Gravendeel-GBM transcriptomic data. Comparison carried out on all IDH-wild-type samples and in a subgroup-specific manner according to Wang's classifier. Size of circle is indicative of R-square value and bold outline represents a $P \leq 0.05$.

B Representative images of differentiation protocol of EPSC into EBS, YS-EBs and iMGL. Scale bar is 50 μm.

C Representative flow cytometry plots for changes in expression of surface markers during differentiation of EPSC to iMGL. Histogram of expression of SOX2 (stem cell marker), PU.1 (early yolk sac marker), P2RY12 (microglia-specific marker) and CD49d (macrophage specific marker) by EPSC, YS-EBs and iMGL are shown.

D Representative flow cytometry plots of expression of p-S6, p-STAT3 and p-NF-κB (p-P65) in iMGL following 4 h of incubation with different culture conditions.

© 2020 The Authors
