# Supplementary figures and images for: Microglia promote glioblastoma via mTOR‐mediated immunosuppression of the tumour microenvironment
Source: EMBO J. 2020 Jun 22;39(15):e103790. doi: 10.15252/embj.2019103790 (PMC7396846; doi:10.15252/embj.2019103790)

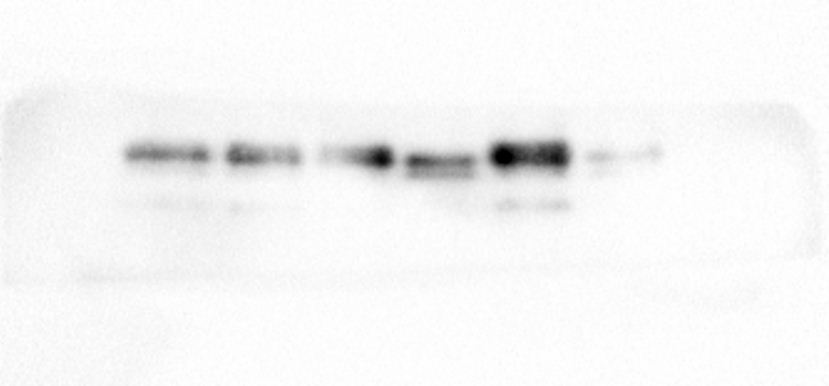

Supplement: Supplementary file 9 — Source Data for Expanded View [file EMBJ-39-e103790-s010.zip › EMBO J-2019-103790R2 Source Files Figure EV1/Figure EV1C/Fig.EV1C_BMDM-4EBP1.JPG]

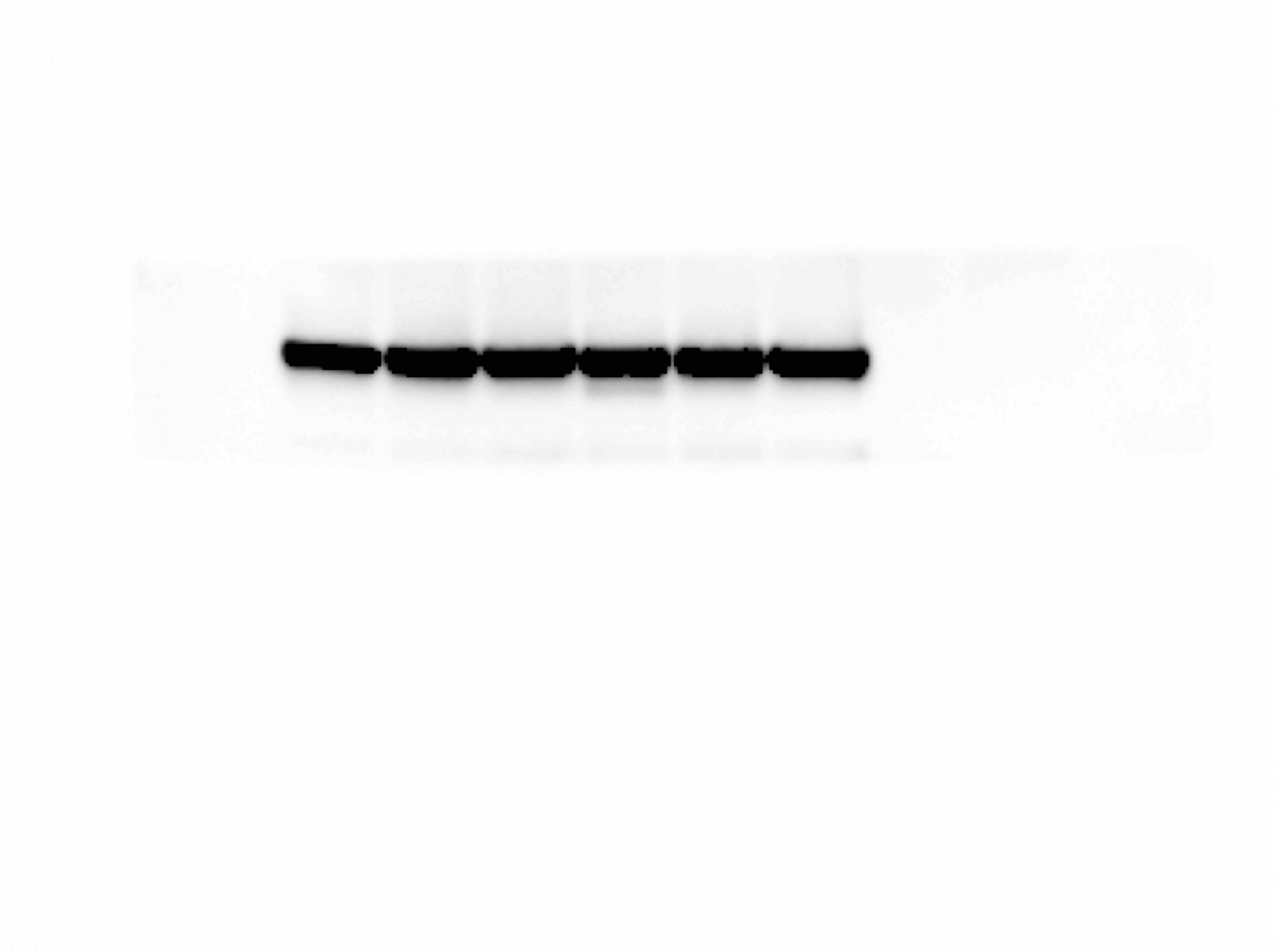

Supplement: Supplementary file 9 — Source Data for Expanded View [file EMBJ-39-e103790-s010.zip › EMBO J-2019-103790R2 Source Files Figure EV1/Figure EV1C/Fig.EV1C_BMDM-AKT for pAKT(S473).jpg]

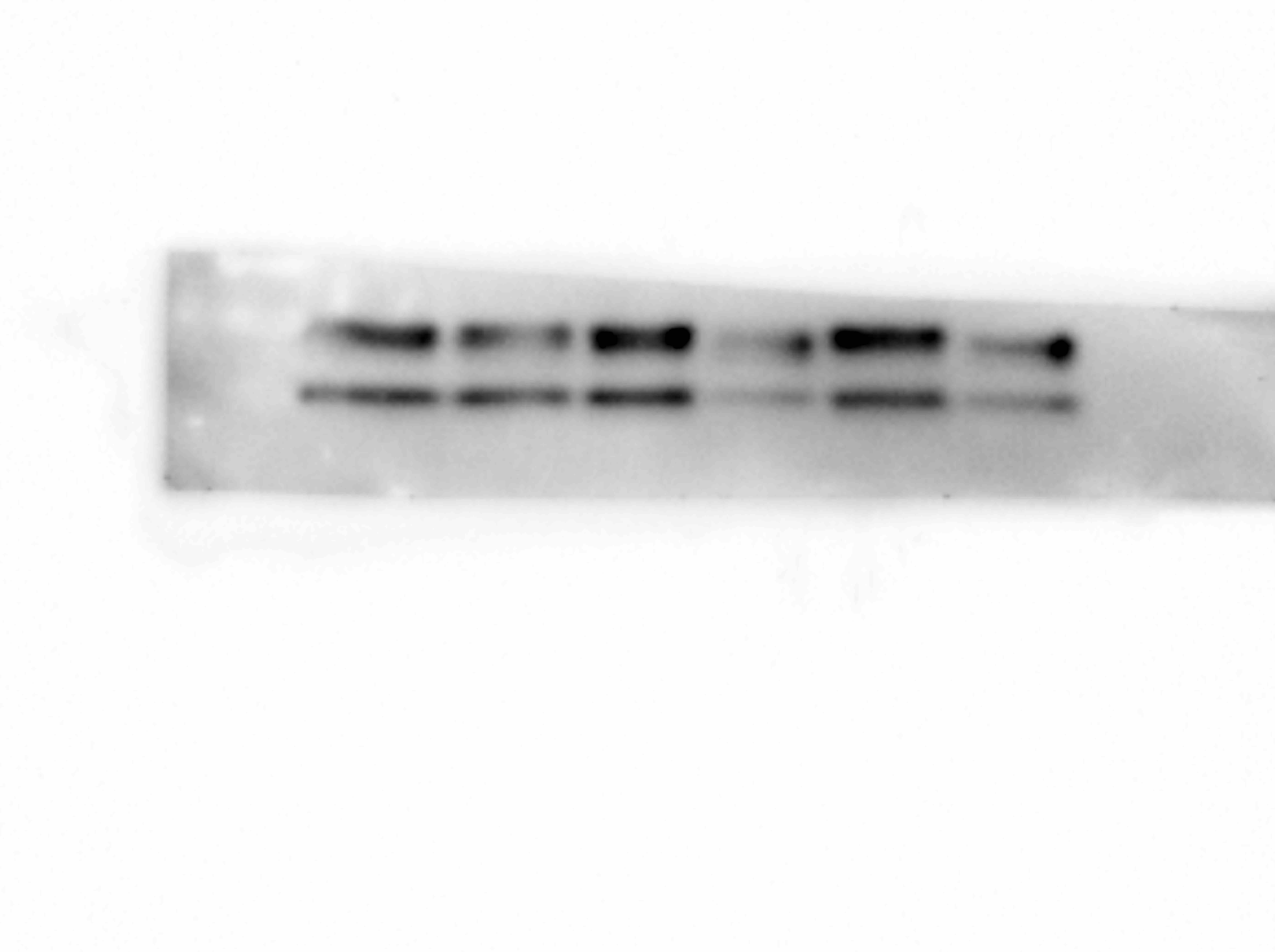

Supplement: Supplementary file 9 — Source Data for Expanded View [file EMBJ-39-e103790-s010.zip › EMBO J-2019-103790R2 Source Files Figure EV1/Figure EV1C/Fig.EV1C_BMDM-p4EBP1.jpg]

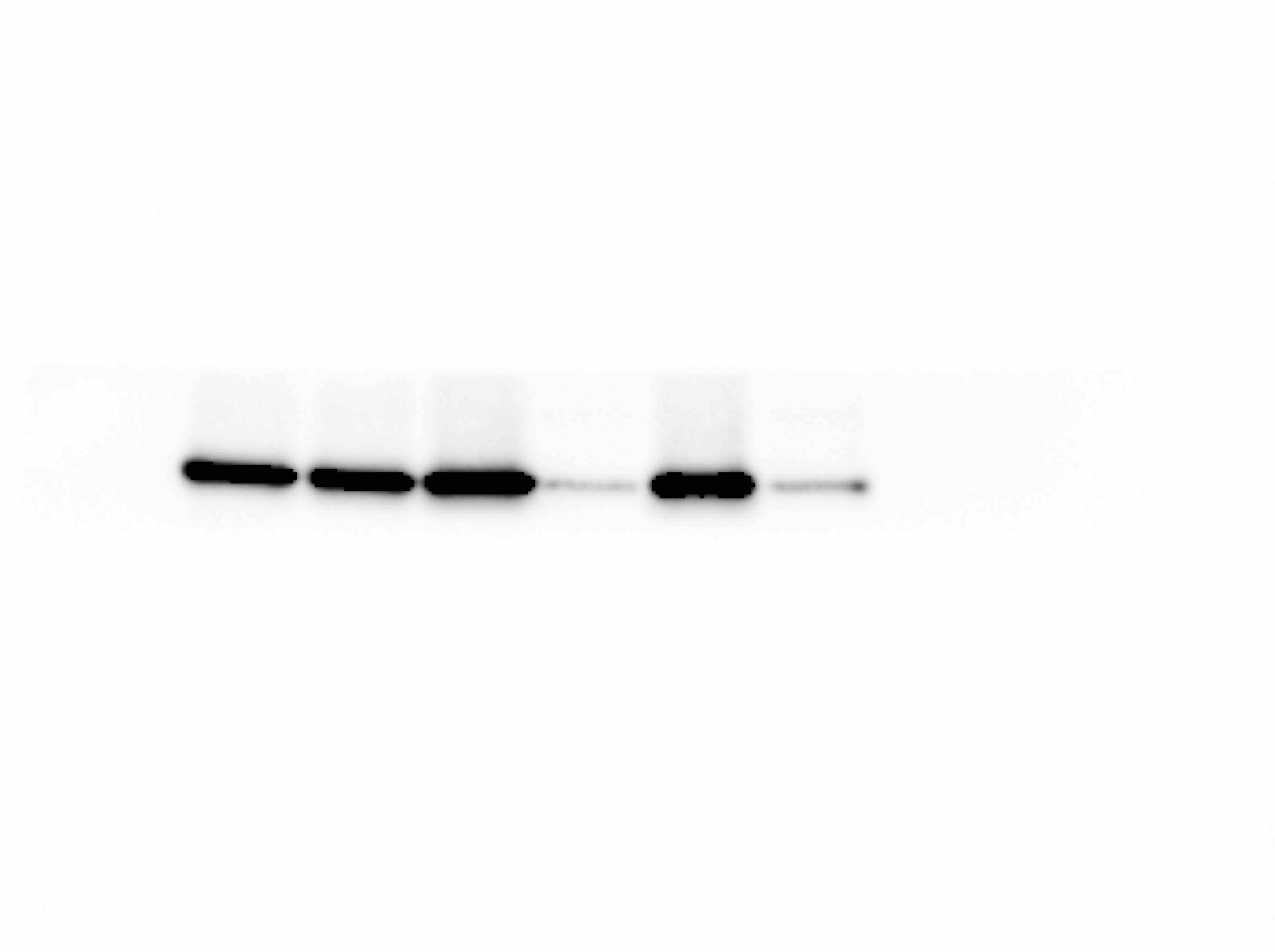

Supplement: Supplementary file 9 — Source Data for Expanded View [file EMBJ-39-e103790-s010.zip › EMBO J-2019-103790R2 Source Files Figure EV1/Figure EV1C/Fig.EV1C_BMDM-pAKT(S473).jpg]

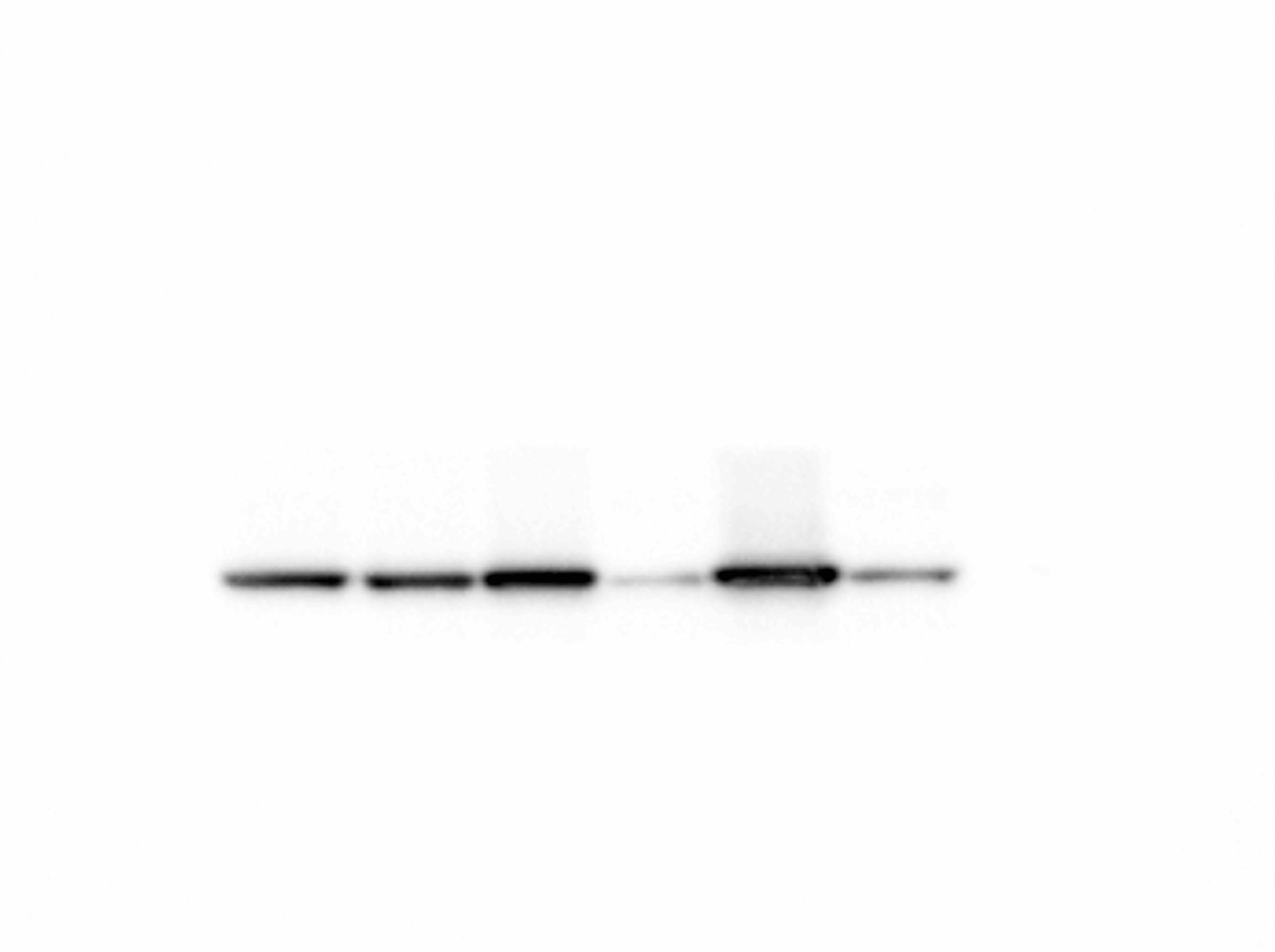

Supplement: Supplementary file 9 — Source Data for Expanded View [file EMBJ-39-e103790-s010.zip › EMBO J-2019-103790R2 Source Files Figure EV1/Figure EV1C/Fig.EV1C_BMDM-pS6.jpg]

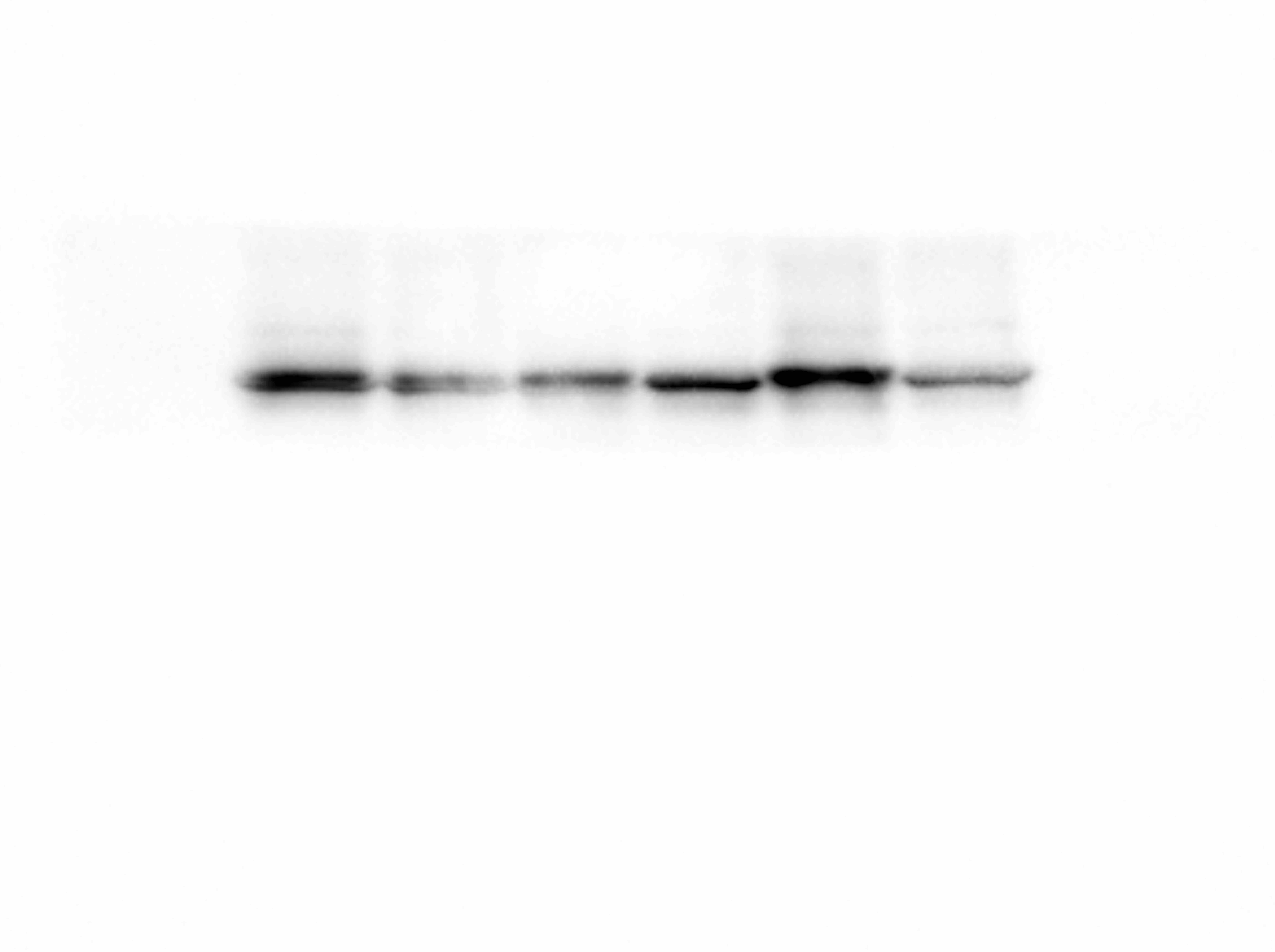

Supplement: Supplementary file 9 — Source Data for Expanded View [file EMBJ-39-e103790-s010.zip › EMBO J-2019-103790R2 Source Files Figure EV1/Figure EV1C/Fig.EV1C_BMDM-S6.jpg]

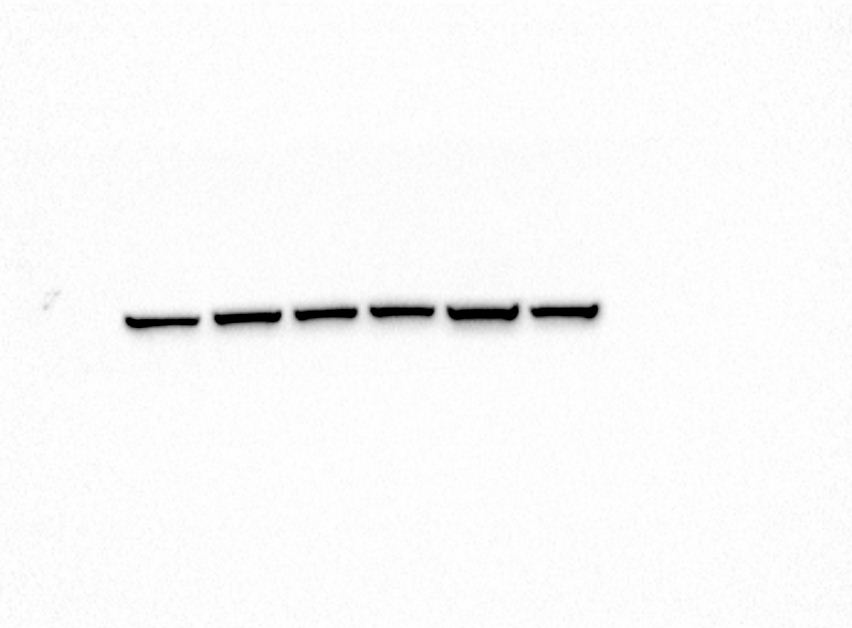

Supplement: Supplementary file 9 — Source Data for Expanded View [file EMBJ-39-e103790-s010.zip › EMBO J-2019-103790R2 Source Files Figure EV1/Figure EV1C/Fig.EV1C_BMDM-vinculin.JPG]

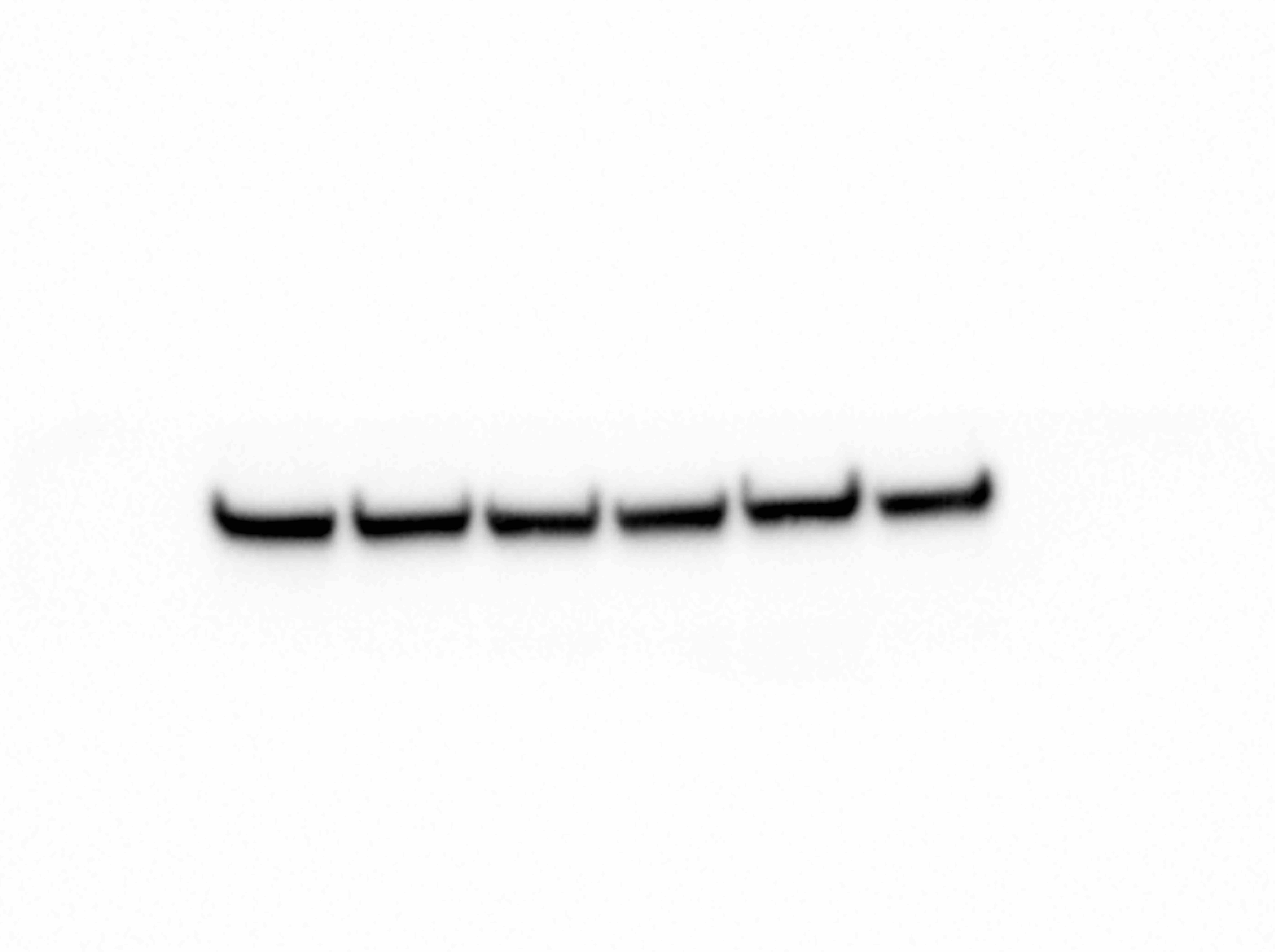

Supplement: Supplementary file 9 — Source Data for Expanded View [file EMBJ-39-e103790-s010.zip › EMBO J-2019-103790R2 Source Files Figure EV1/Figure EV1D/Fig.EV1D_BMDM-AKT for pAKT(T308).jpg]

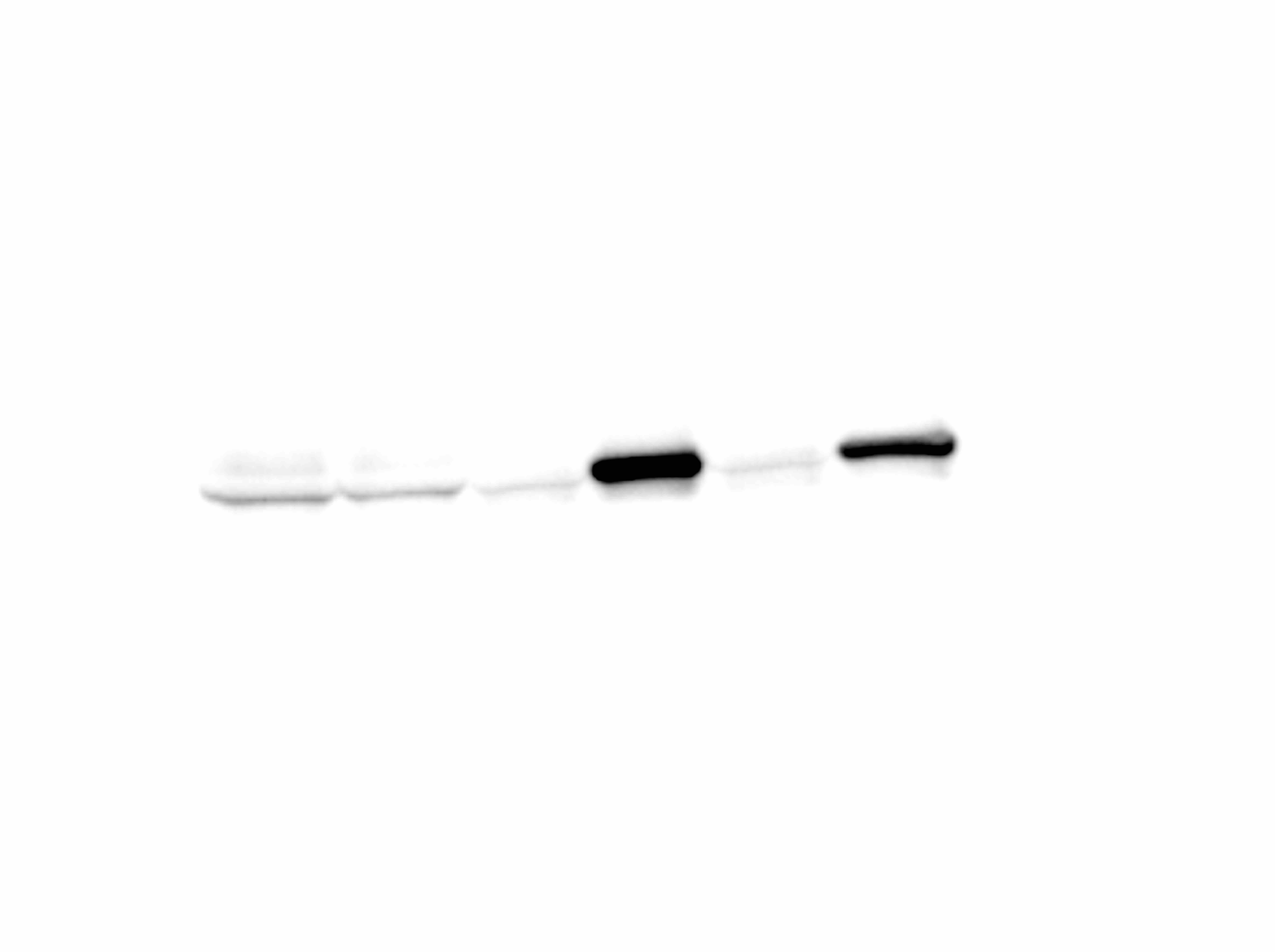

Supplement: Supplementary file 9 — Source Data for Expanded View [file EMBJ-39-e103790-s010.zip › EMBO J-2019-103790R2 Source Files Figure EV1/Figure EV1D/Fig.EV1D_BMDM-pAKT(T308).jpg]

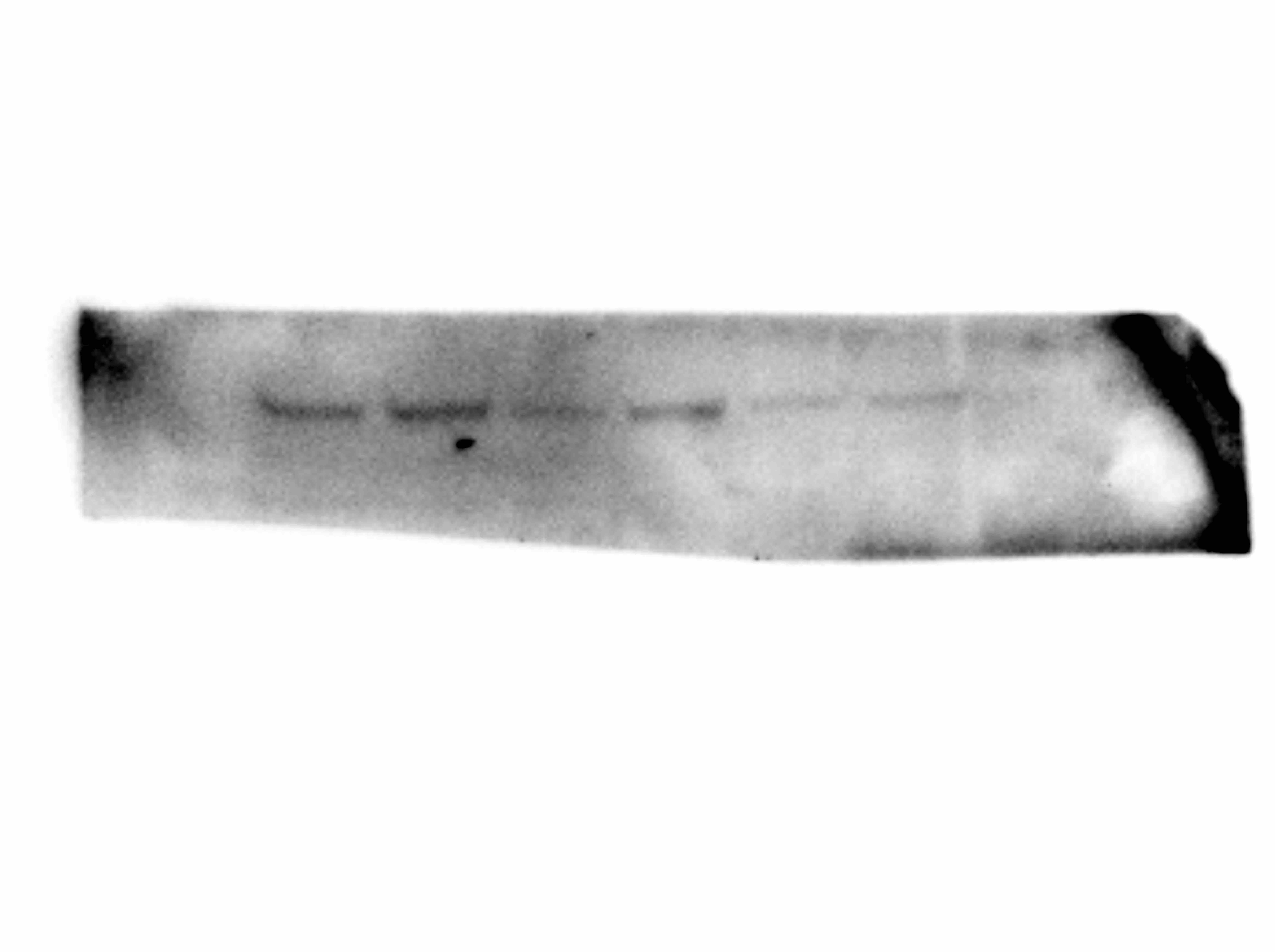

Supplement: Supplementary file 9 — Source Data for Expanded View [file EMBJ-39-e103790-s010.zip › EMBO J-2019-103790R2 Source Files Figure EV1/Figure EV1D/Fig.EV1D_BMDM-Vinculin for pAKT(T308).jpg]

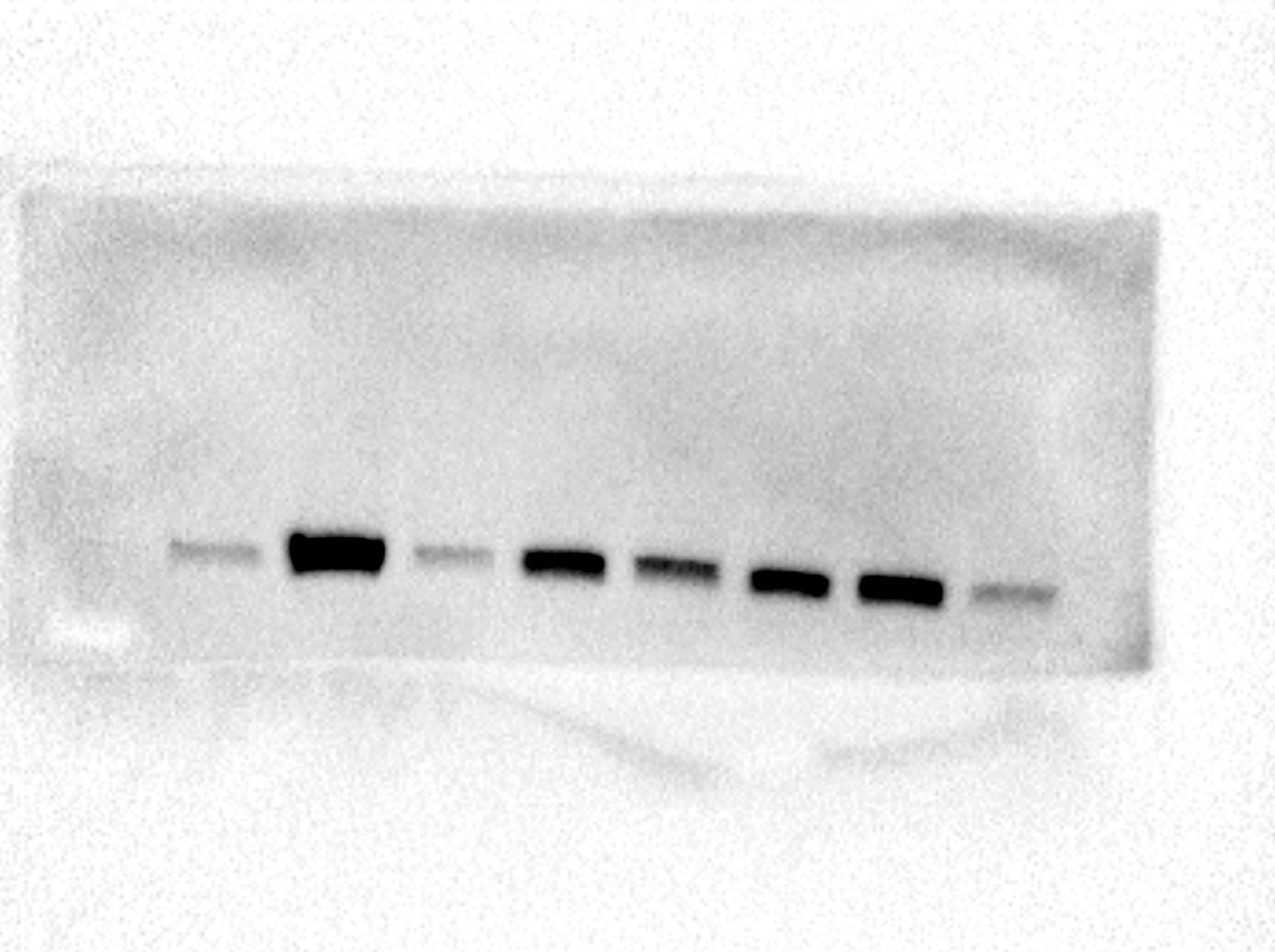

Supplement: Supplementary file 11 — Source Data for Figure 2 [file EMBJ-39-e103790-s009.zip › EMBO J-2019-103790R2 Source Files Figure 2/Fig.2B MG WB pS6/GIC_pS6.jpg]

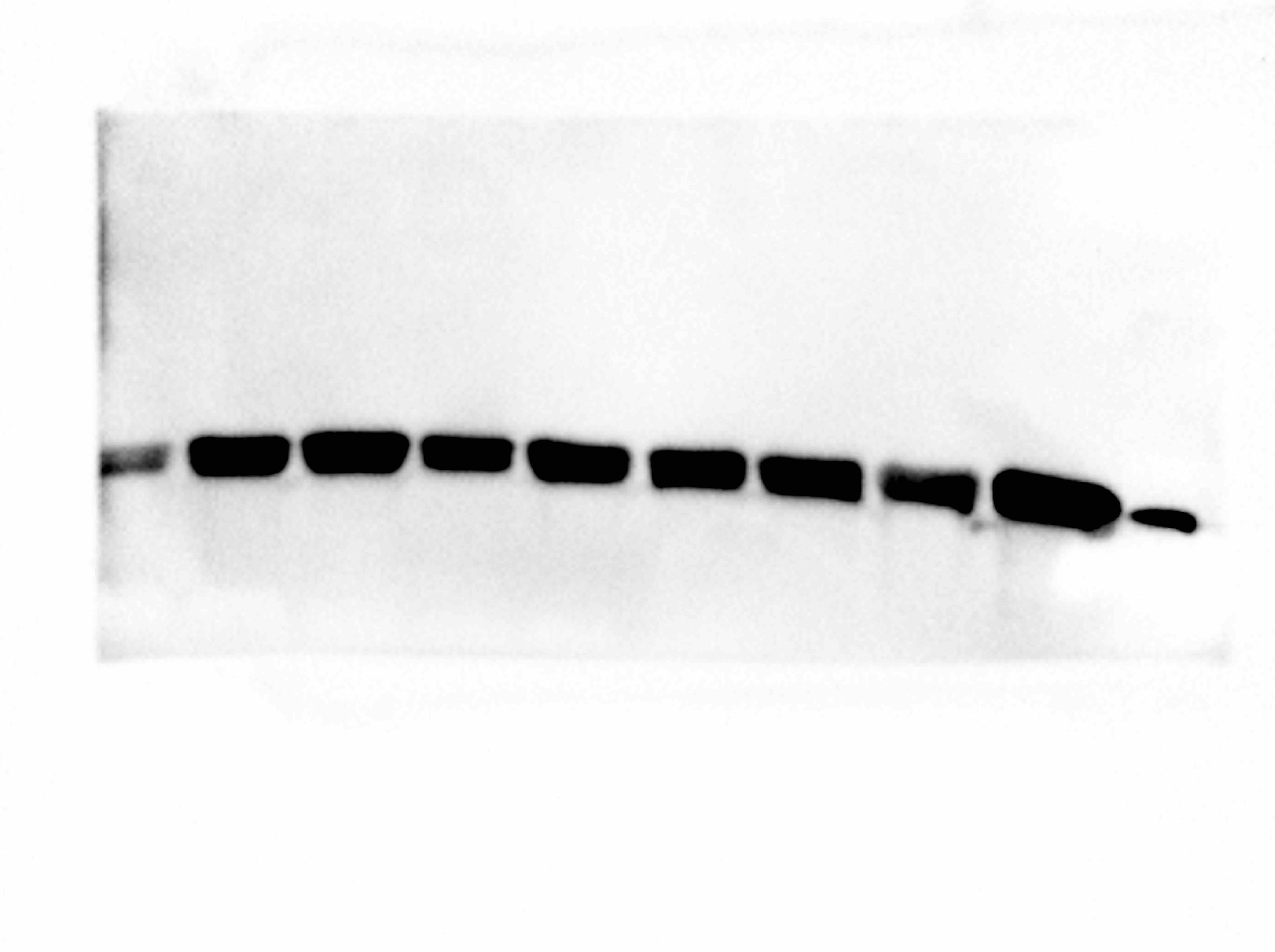

Supplement: Supplementary file 11 — Source Data for Figure 2 [file EMBJ-39-e103790-s009.zip › EMBO J-2019-103790R2 Source Files Figure 2/Fig.2B MG WB pS6/GIC_S6.jpg]

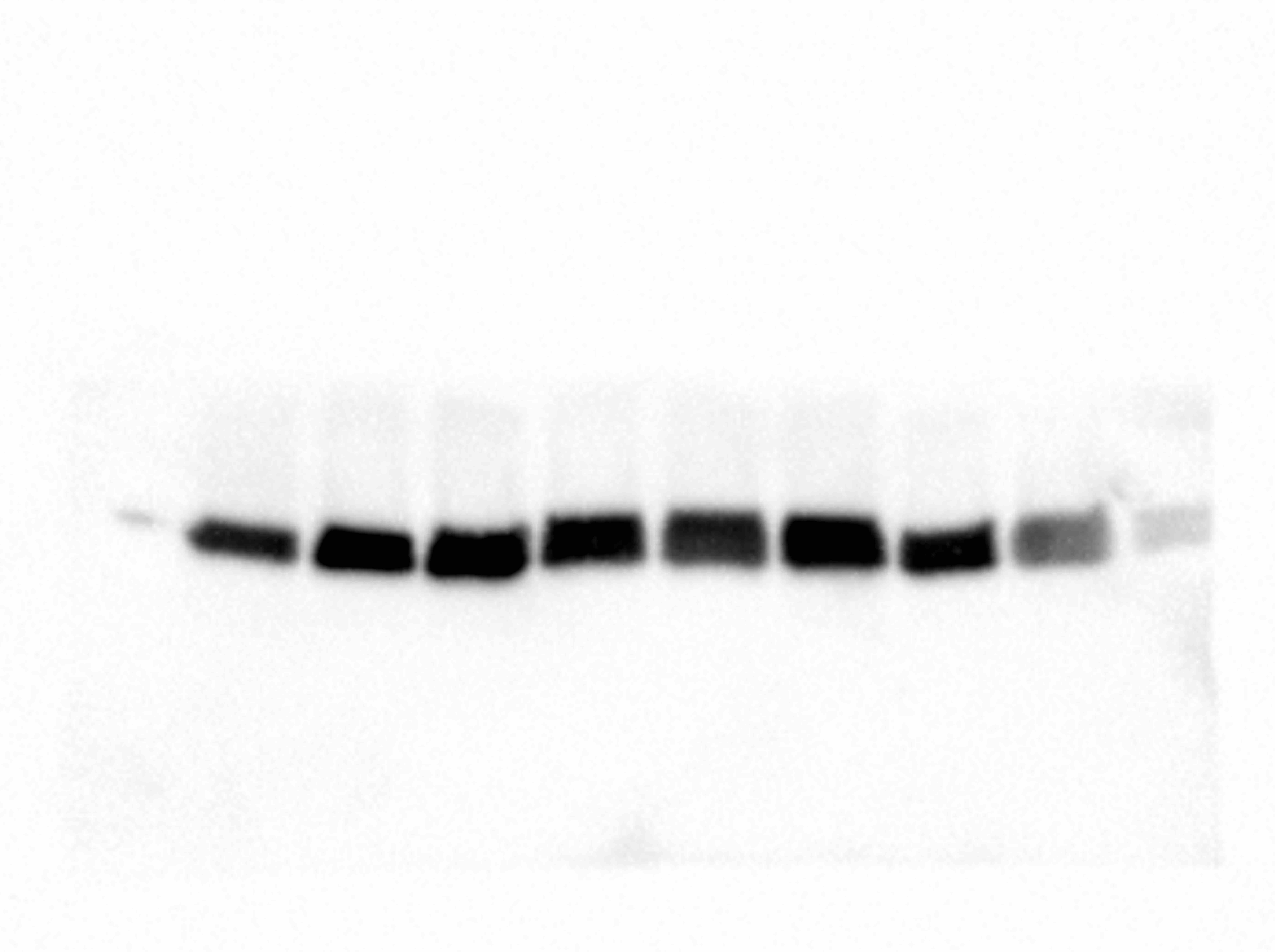

Supplement: Supplementary file 11 — Source Data for Figure 2 [file EMBJ-39-e103790-s009.zip › EMBO J-2019-103790R2 Source Files Figure 2/Fig.2B MG WB pS6/GIC_vinculin.jpg]

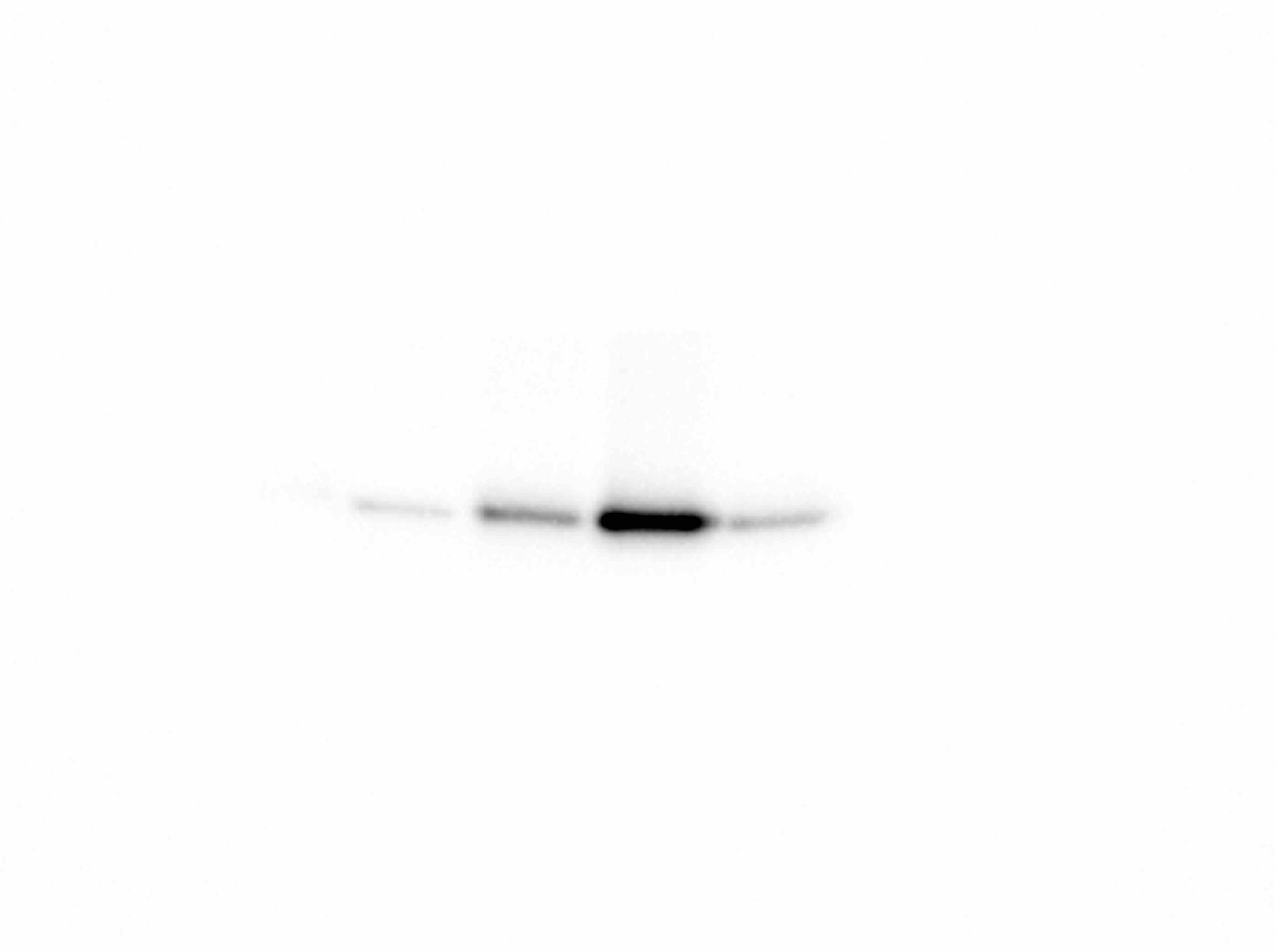

Supplement: Supplementary file 11 — Source Data for Figure 2 [file EMBJ-39-e103790-s009.zip › EMBO J-2019-103790R2 Source Files Figure 2/Fig.2B MG WB pS6/GL261_pS6.jpg]

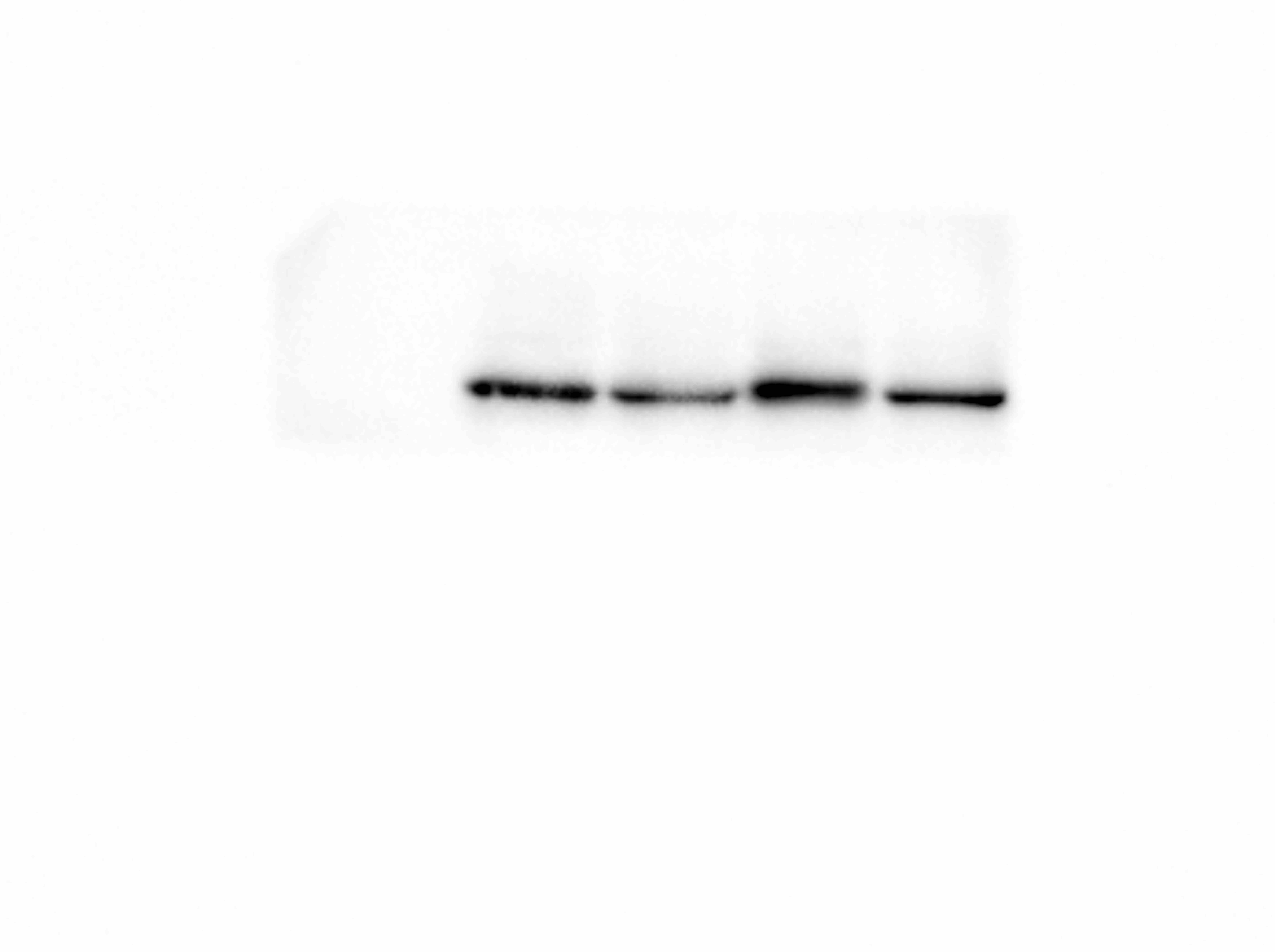

Supplement: Supplementary file 11 — Source Data for Figure 2 [file EMBJ-39-e103790-s009.zip › EMBO J-2019-103790R2 Source Files Figure 2/Fig.2B MG WB pS6/GL261_S6.jpg]

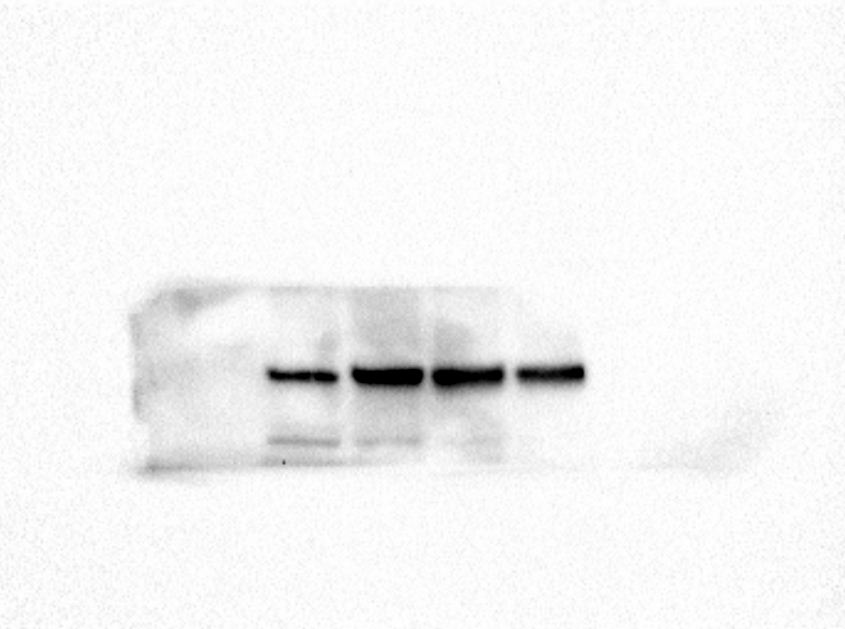

Supplement: Supplementary file 11 — Source Data for Figure 2 [file EMBJ-39-e103790-s009.zip › EMBO J-2019-103790R2 Source Files Figure 2/Fig.2B MG WB pS6/GL261_vinculin.JPG]

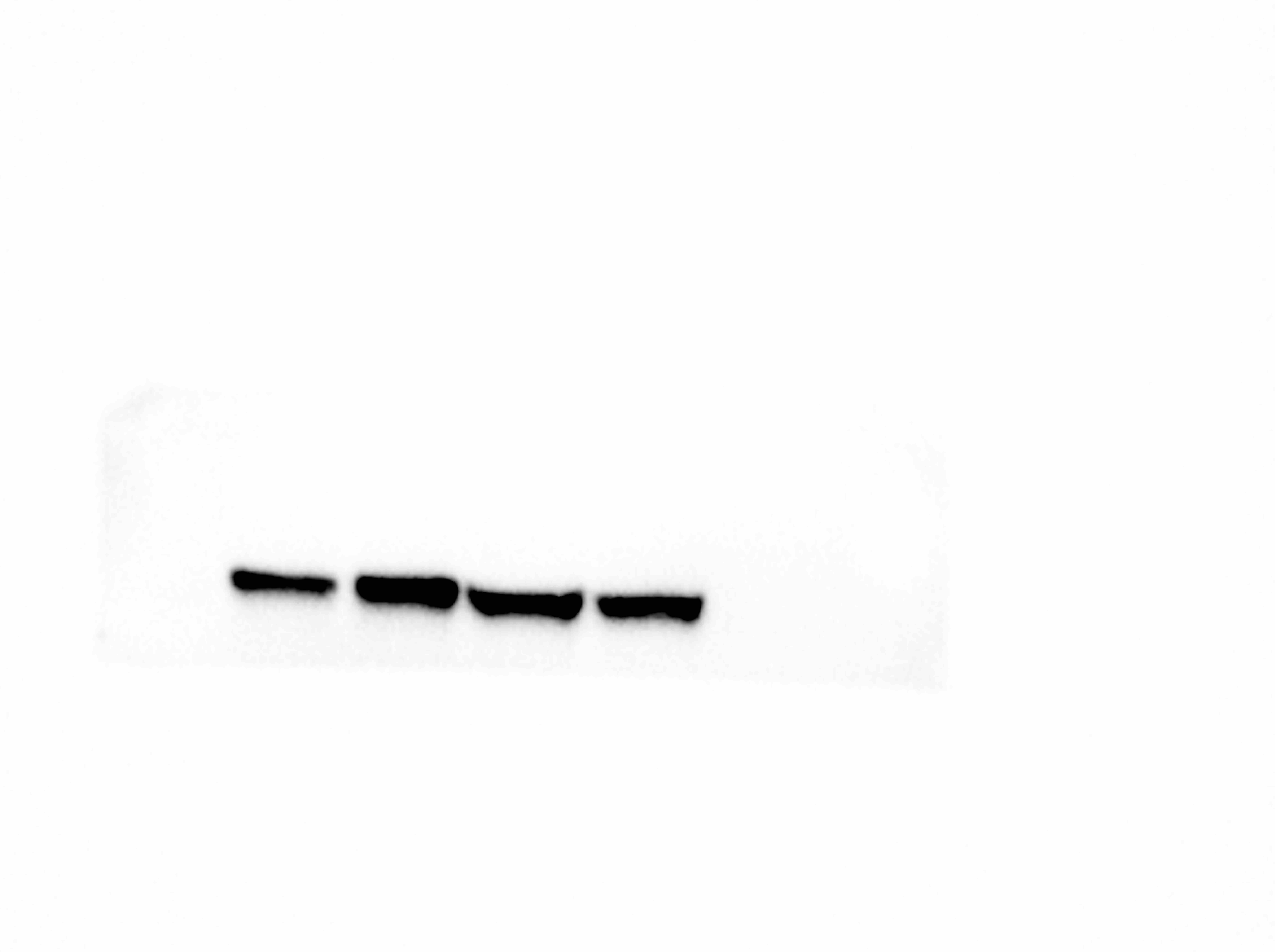

Supplement: Supplementary file 11 — Source Data for Figure 2 [file EMBJ-39-e103790-s009.zip › EMBO J-2019-103790R2 Source Files Figure 2/Fig.2D_MG_WB-p4EBP1/GIC_4EBP1.jpg]

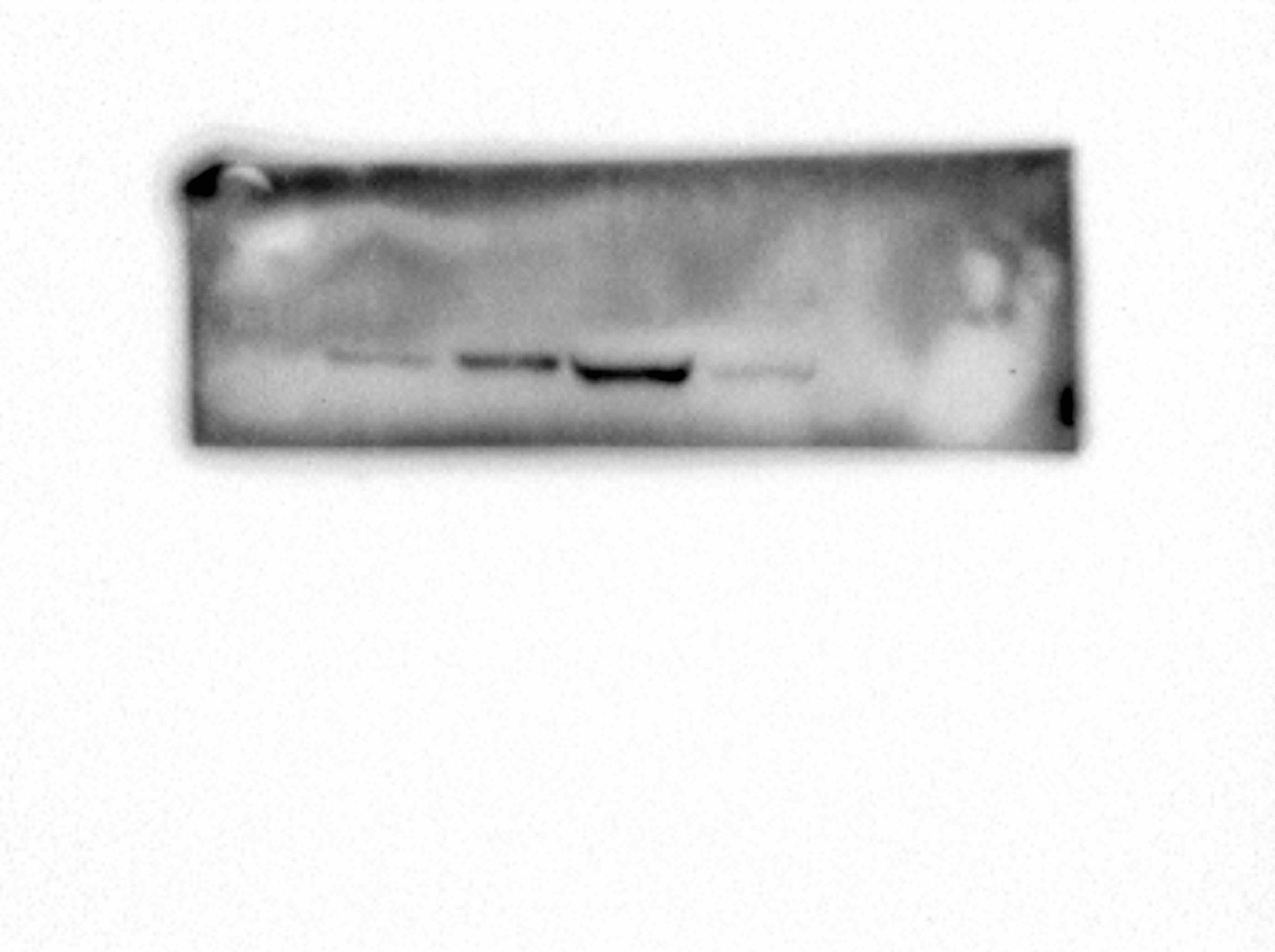

Supplement: Supplementary file 11 — Source Data for Figure 2 [file EMBJ-39-e103790-s009.zip › EMBO J-2019-103790R2 Source Files Figure 2/Fig.2D_MG_WB-p4EBP1/GIC_p4EBP1.jpg]

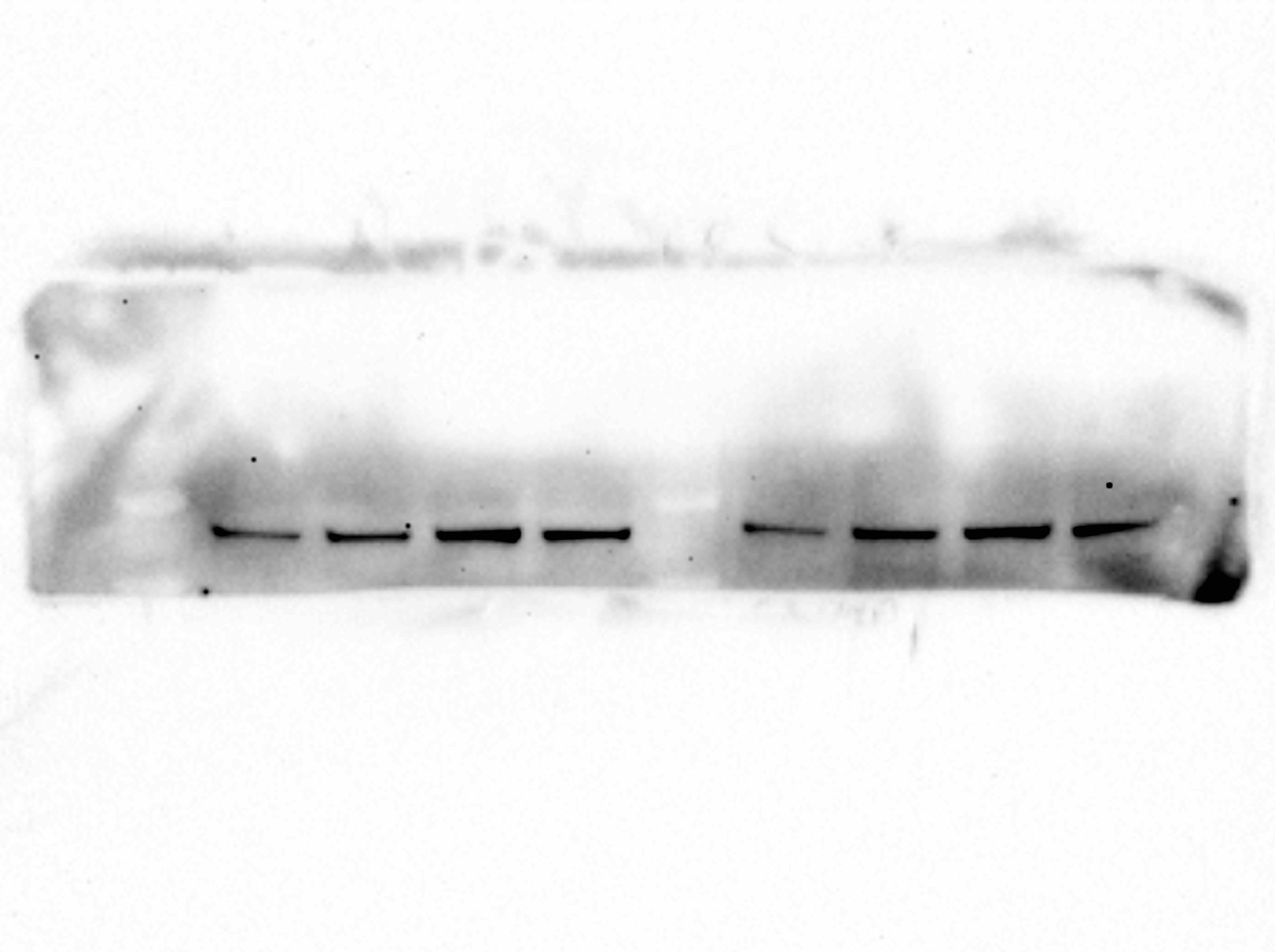

Supplement: Supplementary file 11 — Source Data for Figure 2 [file EMBJ-39-e103790-s009.zip › EMBO J-2019-103790R2 Source Files Figure 2/Fig.2D_MG_WB-p4EBP1/GIC_vinculin (left image).jpg]

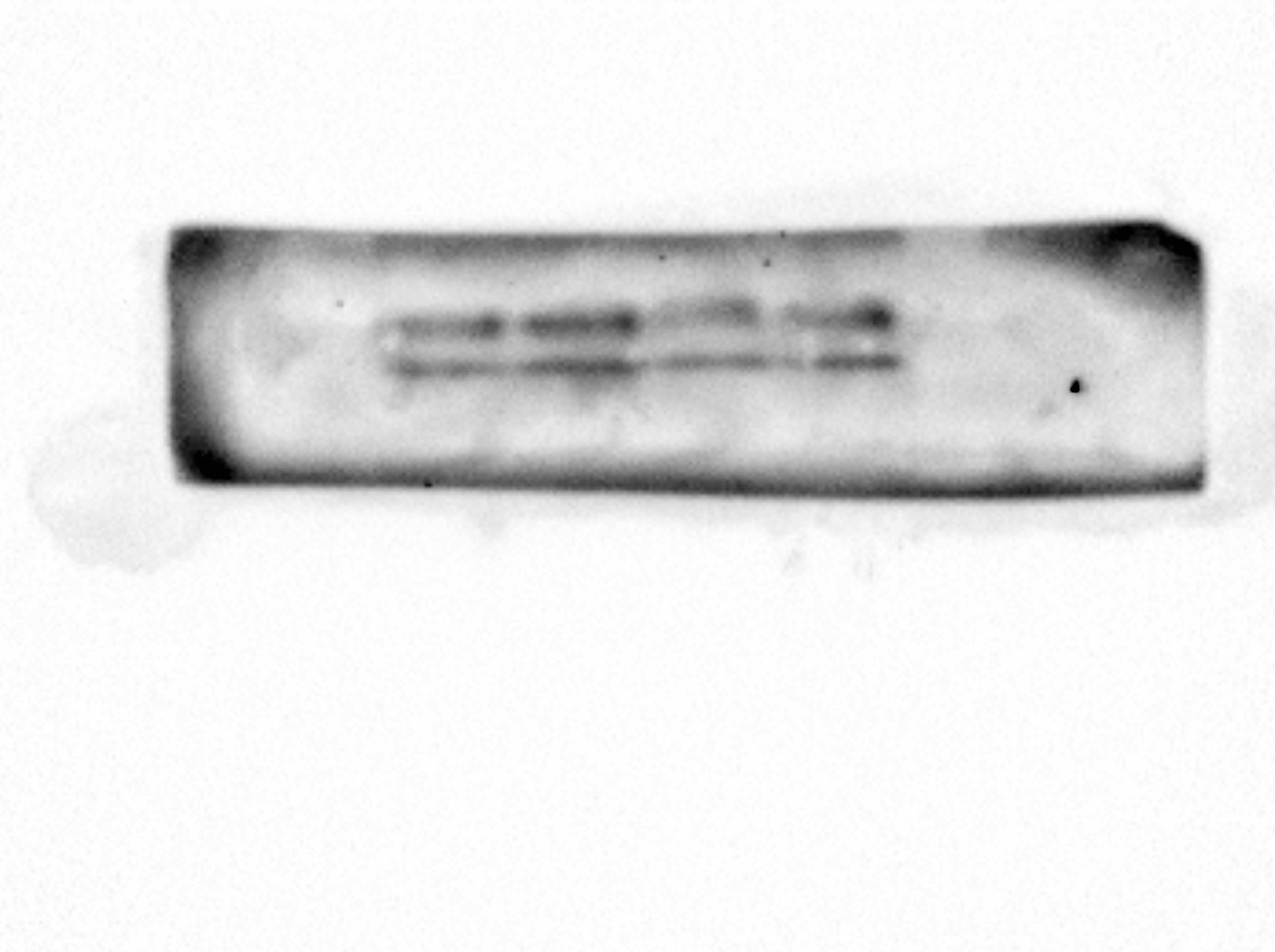

Supplement: Supplementary file 11 — Source Data for Figure 2 [file EMBJ-39-e103790-s009.zip › EMBO J-2019-103790R2 Source Files Figure 2/Fig.2D_MG_WB-p4EBP1/GL261_4EBP1.jpg]

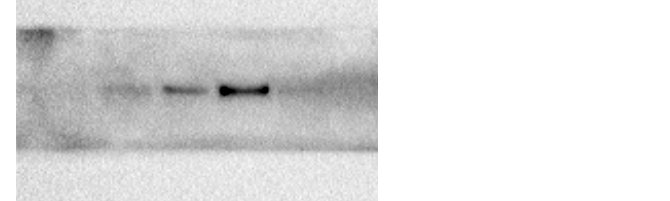

Supplement: Supplementary file 11 — Source Data for Figure 2 [file EMBJ-39-e103790-s009.zip › EMBO J-2019-103790R2 Source Files Figure 2/Fig.2D_MG_WB-p4EBP1/GL261_p4EBP1.png]

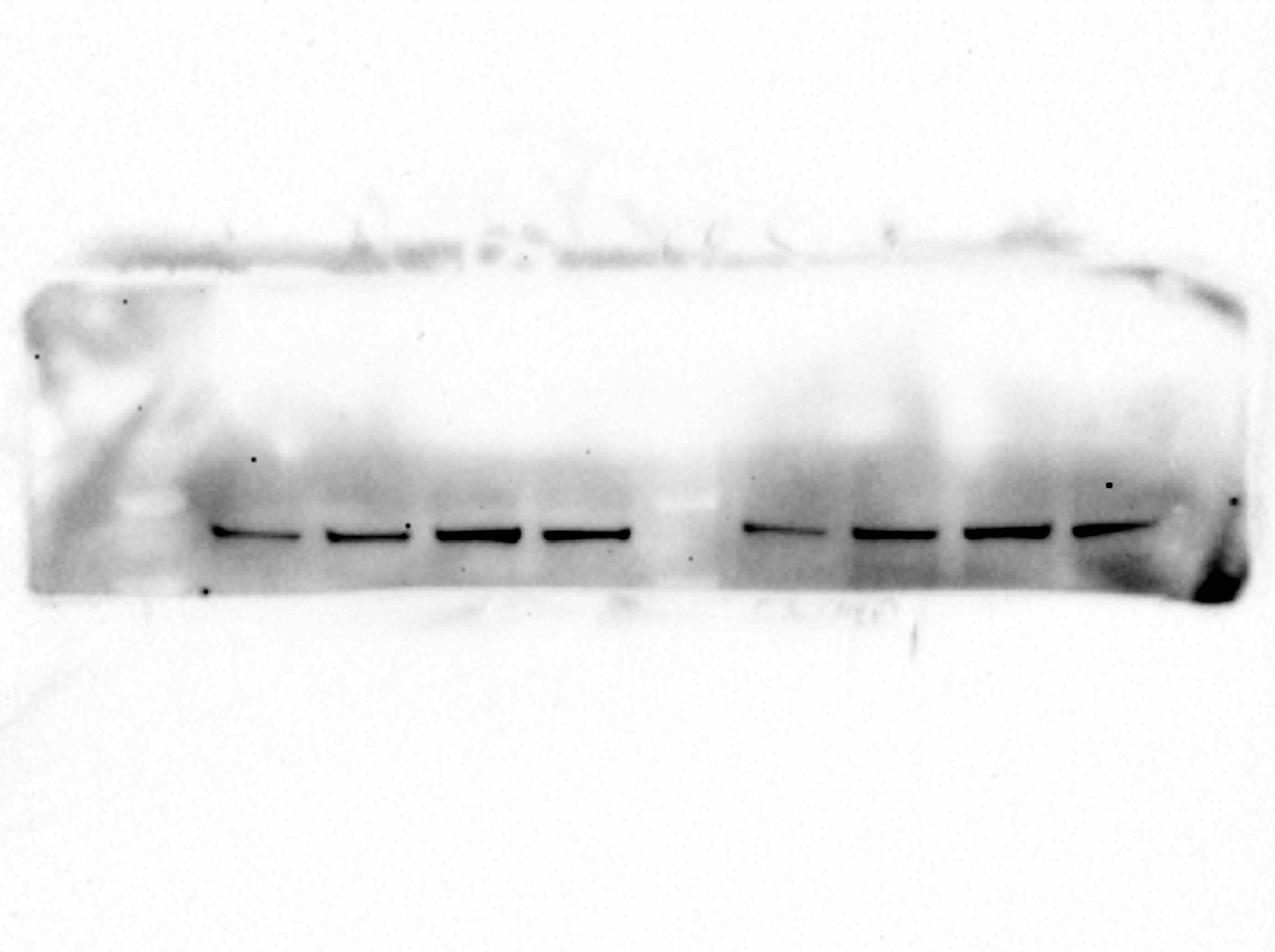

Supplement: Supplementary file 11 — Source Data for Figure 2 [file EMBJ-39-e103790-s009.zip › EMBO J-2019-103790R2 Source Files Figure 2/Fig.2F_MG-WB _pAKT (S473)/GIC_vinculin (right image).jpeg]

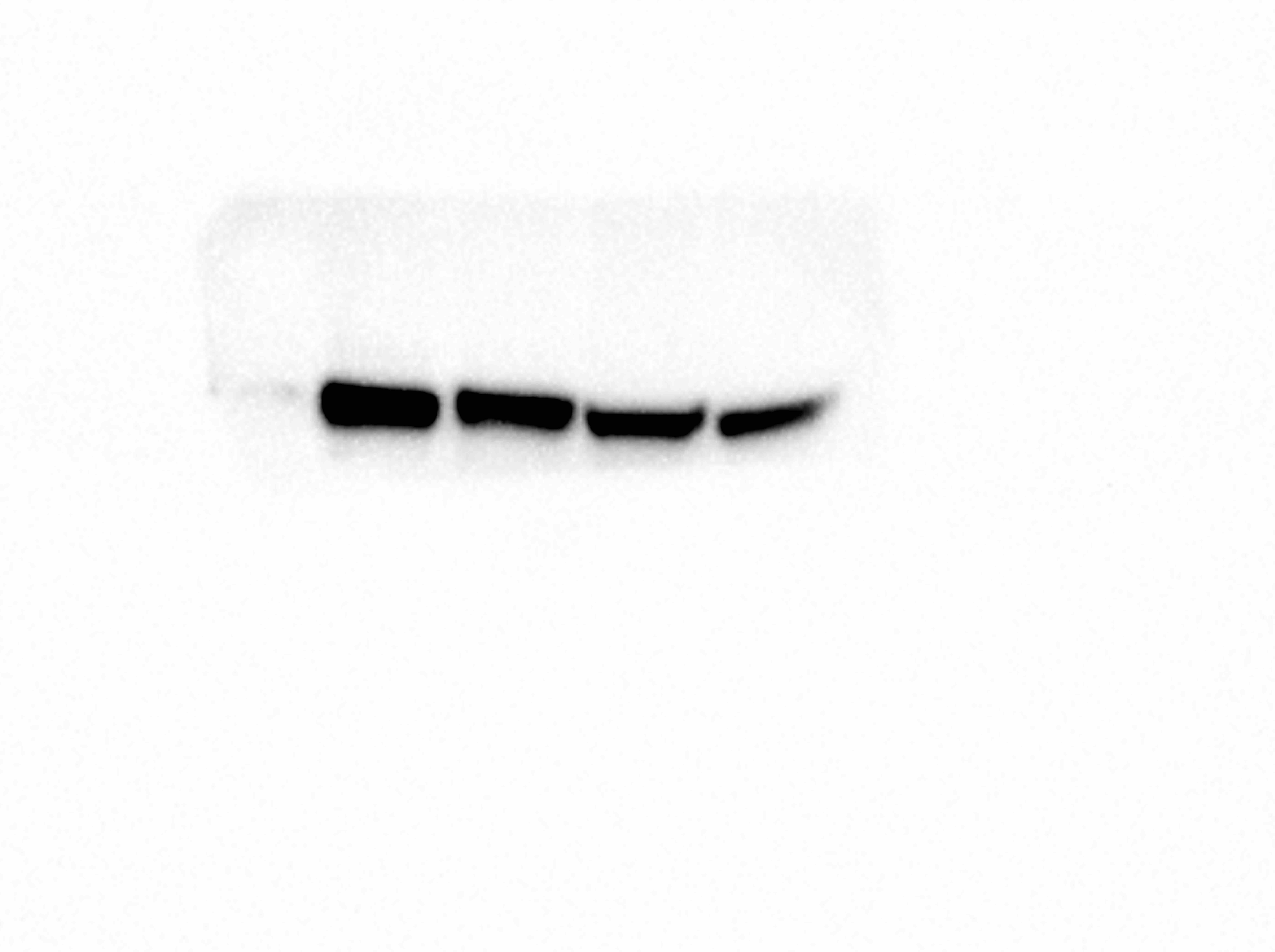

Supplement: Supplementary file 11 — Source Data for Figure 2 [file EMBJ-39-e103790-s009.zip › EMBO J-2019-103790R2 Source Files Figure 2/Fig.2F_MG-WB _pAKT (S473)/GL261_AKT_forpAKT(S473).jpg]

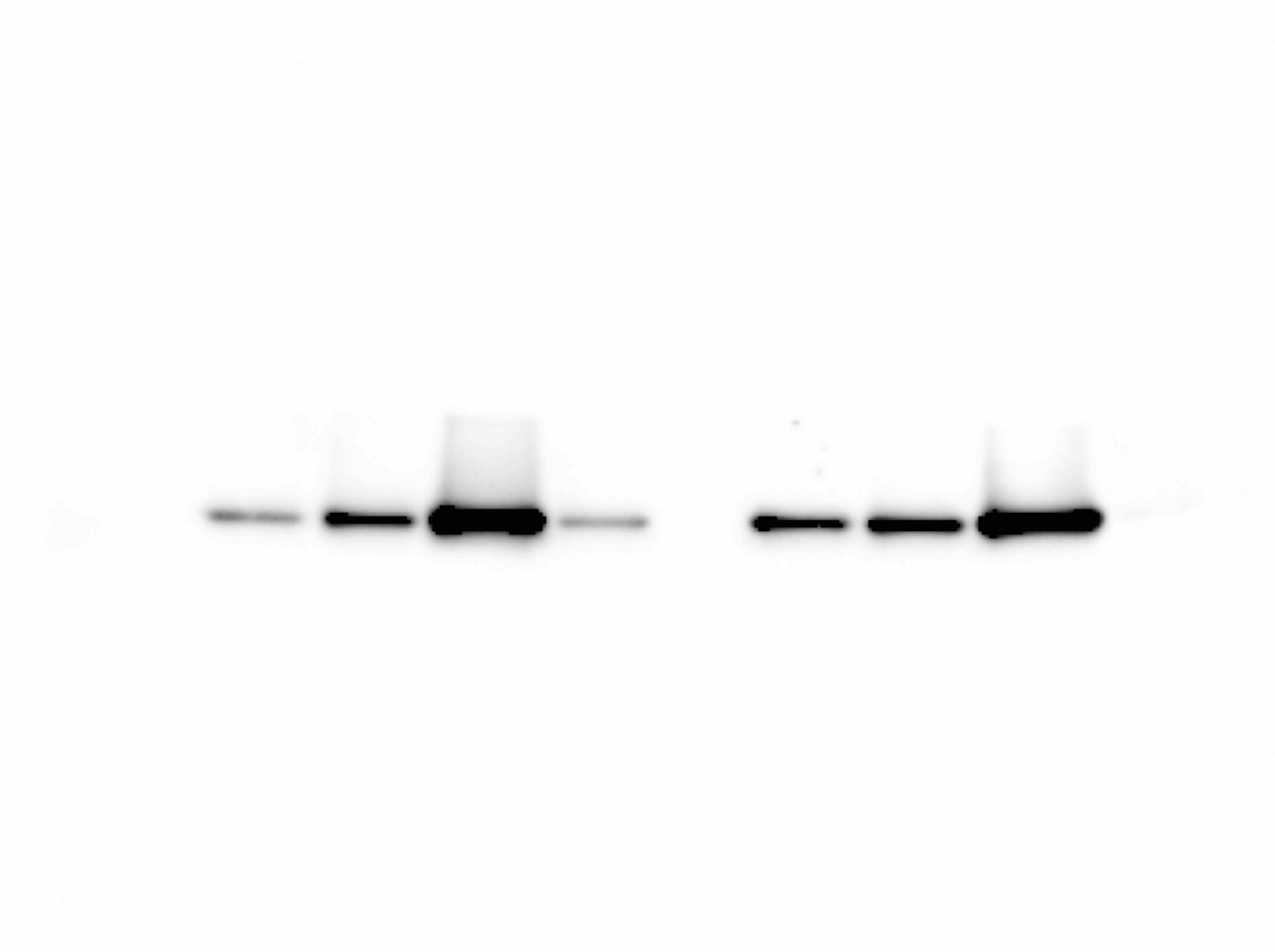

Supplement: Supplementary file 11 — Source Data for Figure 2 [file EMBJ-39-e103790-s009.zip › EMBO J-2019-103790R2 Source Files Figure 2/Fig.2F_MG-WB _pAKT (S473)/GL261_pAKT(S473) (right image).jpg]

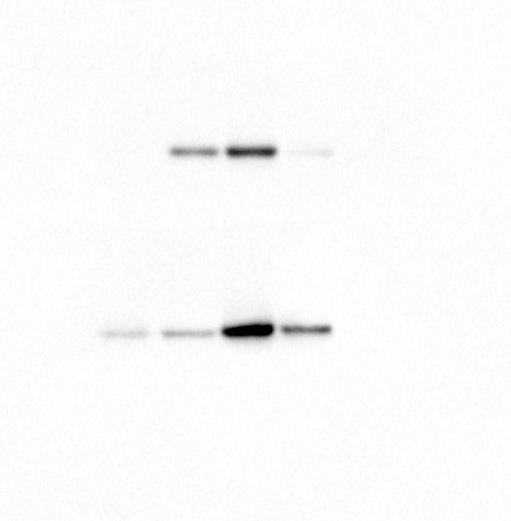

Supplement: Supplementary file 11 — Source Data for Figure 2 [file EMBJ-39-e103790-s009.zip › EMBO J-2019-103790R2 Source Files Figure 2/Fig.2H_MG_WB-pAKT (T308)/GIC_pAKT (T308) (bottom gel).tif]

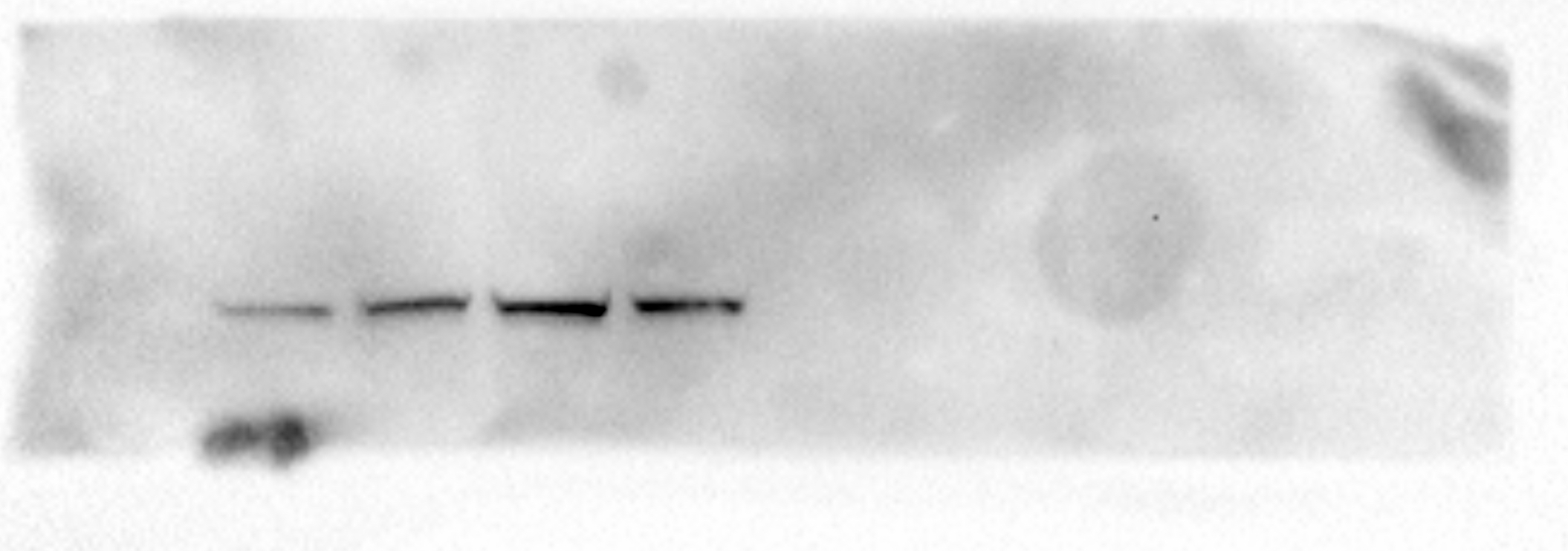

Supplement: Supplementary file 11 — Source Data for Figure 2 [file EMBJ-39-e103790-s009.zip › EMBO J-2019-103790R2 Source Files Figure 2/Fig.2H_MG_WB-pAKT (T308)/GIC_vinculin.jpg]

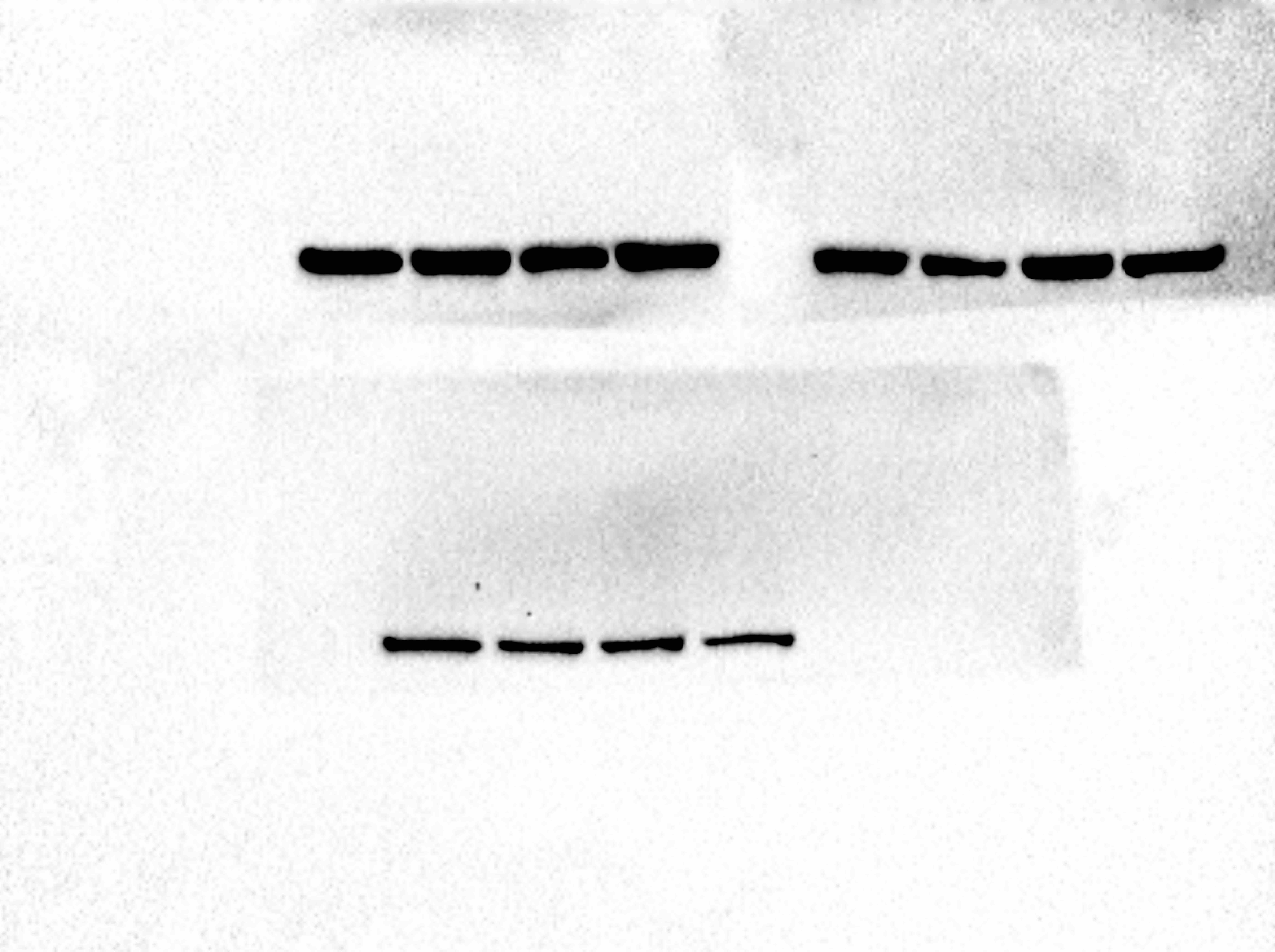

Supplement: Supplementary file 11 — Source Data for Figure 2 [file EMBJ-39-e103790-s009.zip › EMBO J-2019-103790R2 Source Files Figure 2/Fig.2H_MG_WB-pAKT (T308)/GL261_AKT (bottom image).jpg]

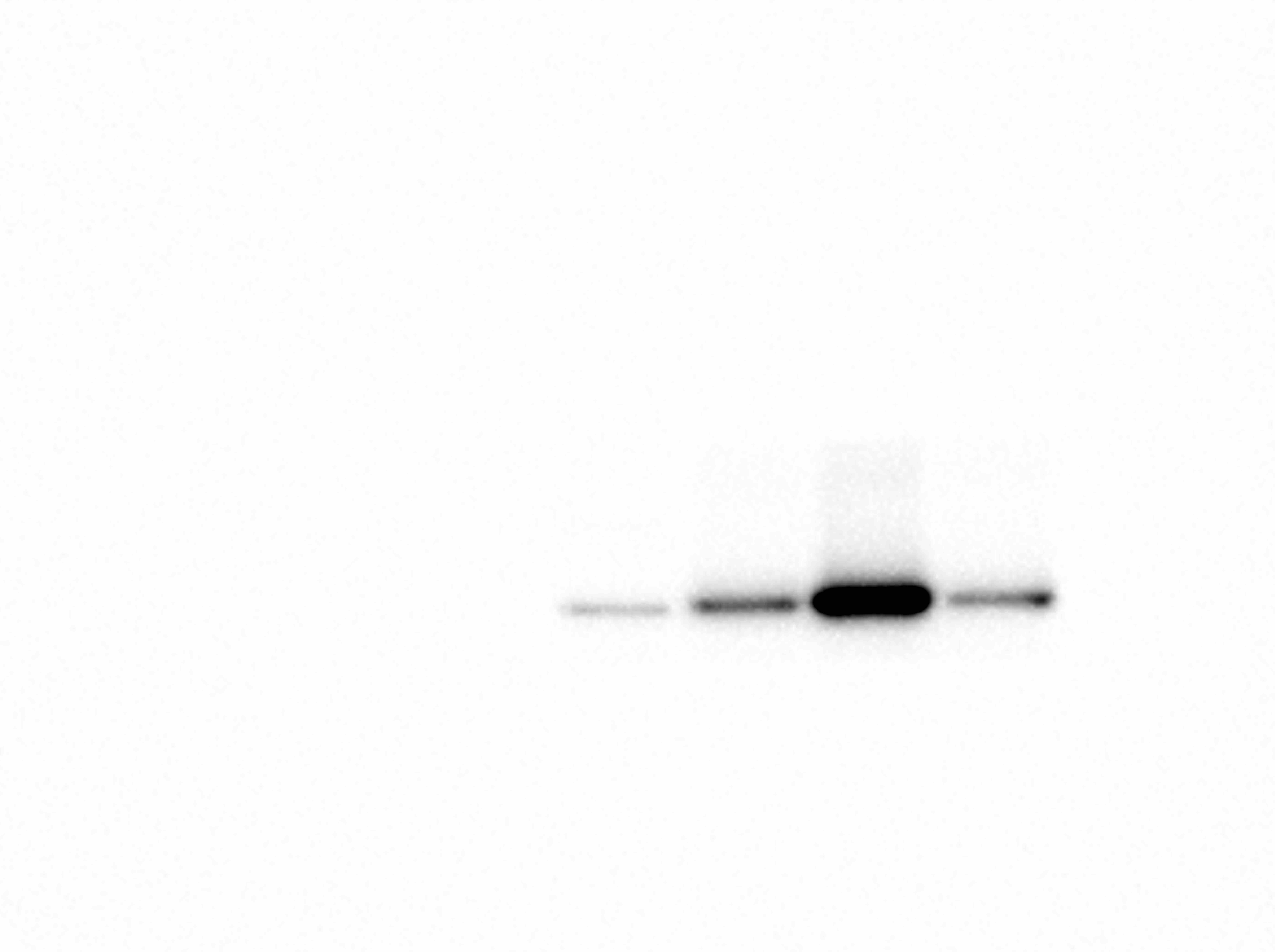

Supplement: Supplementary file 11 — Source Data for Figure 2 [file EMBJ-39-e103790-s009.zip › EMBO J-2019-103790R2 Source Files Figure 2/Fig.2H_MG_WB-pAKT (T308)/GL261_pAKT(T308).jpg]

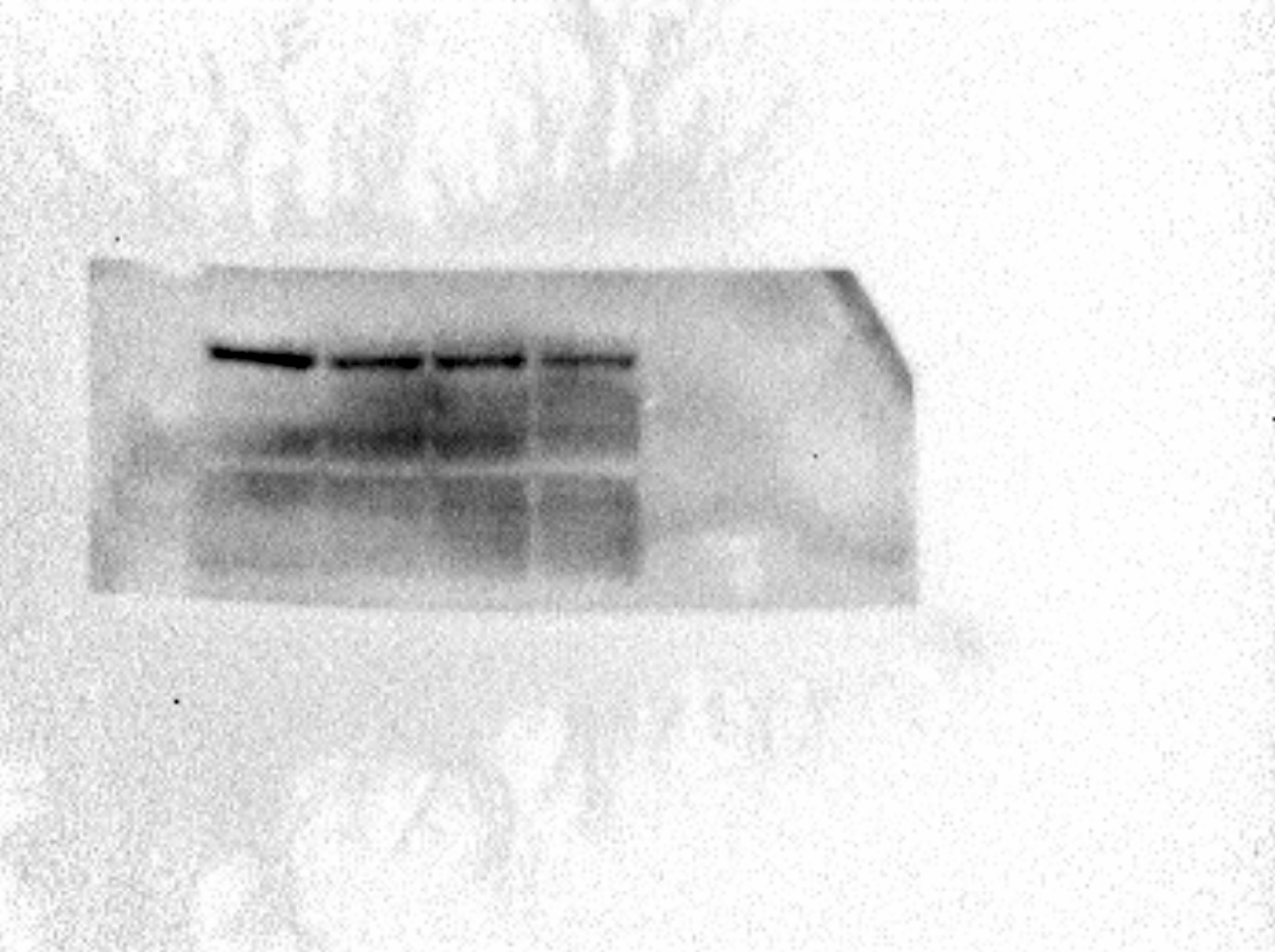

Supplement: Supplementary file 11 — Source Data for Figure 2 [file EMBJ-39-e103790-s009.zip › EMBO J-2019-103790R2 Source Files Figure 2/Fig.2H_MG_WB-pAKT (T308)/GL261_vinculin_n=2.jpg]
